# HNEAP Regulates Necroptosis of Cardiomyocytes by Suppressing the m^5^C Methylation of Atf7 mRNA

**DOI:** 10.1002/advs.202304329

**Published:** 2023-10-23

**Authors:** Kai Wang, Fu‐Hai Li, Lu‐Yu Zhou, Xue‐Mei Zhao, Xiang‐Qian Gao, Cui‐Yun Liu, Xin‐Min Li, Xin‐Zhe Chen, Yan Zhao, Xue‐Li Cheng, Rui‐Quan Wang, Rui‐Feng Li, Yu‐Hui Zhang, Fei Gao, Jin‐Wei Tian, Kun Wang

**Affiliations:** ^1^ Institute for Translational Medicine The Affiliated Hospital of Qingdao University College of Medicine Qingdao University Qingdao 266021 China; ^2^ Department of Cardiology The Affiliated Hospital of Qingdao University Qingdao 266021 China; ^3^ Department of Pharmacy College of Biology Hunan University Changsha Hunan 410082 China; ^4^ State Key Laboratory of Cardiovascular Disease Heart Failure Center Fuwai Hospital National Center for Cardiovascular Diseases Chinese Academy of Medical Sciences, Peking Union Medical College Beijing 100037 China; ^5^ Department of Pathology Binzhou Medical University Hospital Binzhou 256603 China; ^6^ Department of Cardiology Beijing Anzhen Hospital Capital Medical University Beijing 100029 China; ^7^ Department of Cardiology The Second Affiliated Hospital of Harbin Medical University Harbin 150086 China

**Keywords:** ATF7, cardiomyocyte necroptosis, CHMP2A, HNEAP, m^5^C methylation

## Abstract

PIWI‐interacting RNAs (piRNAs) are highly expressed in various cardiovascular diseases. However, their role in cardiomyocyte death caused by ischemia/reperfusion (I/R) injury, especially necroptosis, remains elusive. In this study, a heart necroptosis‐associated piRNA (HNEAP) is found that regulates cardiomyocyte necroptosis by targeting DNA methyltransferase 1 (DNMT1)‐mediated 5‐methylcytosine (m^5^C) methylation of the activating transcription factor 7 (Atf7) mRNA transcript. HNEAP expression level is significantly elevated in hypoxia/reoxygenation (H/R)‐exposed cardiomyocytes and I/R‐injured mouse hearts. Loss of HNEAP inhibited cardiomyocyte necroptosis and ameliorated cardiac function in mice. Mechanistically, HNEAP directly interacts with DNMT1 and attenuates m^5^C methylation of the Atf7 mRNA transcript, which increases Atf7 expression level. ATF7 can further downregulate the transcription of Chmp2a, an inhibitor of necroptosis, resulting in the reduction of Chmp2a level and the progression of cardiomyocyte necroptosis. The findings reveal that piRNA‐mediated m^5^C methylation is involved in the regulation of cardiomyocyte necroptosis. Thus, the HNEAP‐DNMT1‐ATF7‐CHMP2A axis may be a potential target for attenuating cardiac injury caused by necroptosis in ischemic heart disease.

## Graphical abstract

HNEAP regulates cardiomyocyte necroptosis by targeting DNMT1‐mediated 5‐methylcytosine (m^5^C) methylation of the Atf7 mRNA transcript. Mechanistically, HNEAP interacts with DNMT1 and attenuates m^5^C methylation of the Atf7 mRNA transcript, which increases Atf7 expression level. ATF7 further reduces the transcription of Chmp2a, an inhibitor of necroptosis, resulting in the progression of cardiomyocyte necroptosis.

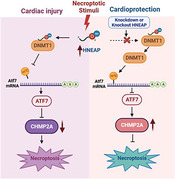

## 1 Introduction

Cardiovascular diseases (CVDs) are the major causes for mortality and morbidity in both developing and developed countries.^[^ ^1^ ^]^ The prevalence of CVDs continues to increase with a trend of early onset in younger individuals.^[^ ^2^ ^]^ Myocardial reperfusion injury is a common cardiac injury encountered in clinical settings. Restoration of blood supply to the ischemic tissue leads to further cardiomyocyte injury in the reperfusion area, such as a severe inflammatory response, cell death, and oxidative stress injury.^[^ ^3^, ^4^ ^]^

For a long time, necrosis was considered to be a passive mechanism of cell death caused by physical or chemical damage factors, hypoxia, malnutrition, and other pathological factors. Programmed necrosis or necroptosis is a newly discovered form of cell death.^[^ ^5^, ^6^ ^]^ Unlike apoptosis and pyroptosis, necroptosis is similar to traditional necrosis, such as rupture of the plasma membrane, which causes an inflammatory response.^[^ ^7^ ^]^ The production and accumulation of pro‐inflammatory cytokines, destruction of biofilms, and release of intracellular damage‐related molecules are closely related to the occurrence and development of necroptosis.^[^ ^8^ ^]^

Necroptosis is regulated by a sophisticated cellular signaling network.^[^ ^9^ ^]^ Under the induction of TNFα, RIPK1 and RIPK3 can phosphorylate each other to form necrosomes.^[^ ^10^ ^]^ RIPK1 and RIPK3 are phosphorylated to facilitate kinase activity, and mixed lineage kinase domain‐like protein (MLKL) is phosphorylated and activated, which further mediates cell necroptosis.^[^ ^10^ ^]^ Necroptosis is relevant in ischemic injury and neurodegenerative diseases, and it has become the focus of cell death research in recent years.^[^ ^11^, ^12^ ^]^

As a hotspot in the field of CVD research, an increasing number of heart‐specific ncRNAs have been shown to be involved in the regulation of heart development and the occurrence and development of various heart diseases.^[^ ^13^ ^]^ PIWI‐interacting RNAs (piRNAs) constitute a new class of non‐coding RNA discovered in recent years; they are ≈24–32 nt in length. However, the specific functions of piRNAs in heart disease remain unclear. The m^5^C modification is characterized by the addition of a methyl group to the fifth carbon atom of cytosine. M^5^C mRNA is involved in mRNA export, maintenance of stability, and translation.^[^ ^14^, ^15^, ^16^ ^]^ DNMT1,2 are crucial methylation regulators.^[^ ^17^, ^18^ ^]^ However, the mechanism by which the m^5^C modification module is dysregulated in heart diseases remains unclear.

In this study, we demonstrated that HNEAP is a key regulator of cardiomyocyte necroptosis during myocardial reperfusion damage. HNEAP intermediates cardiomyocyte necroptosis by directly interacting with DNMT1 and reducing its RNA 5‐methylcytosine (m^5^C) methylation activity, which leads to the upregulation of Atf7 expression and a decrease in the level of ATF7‐antagonistic necroptosis inhibitory factor CHMP2A. Our study revealed that HNEAP‐mediated RNA m^5^C modification significantly contributes to cardiomyocyte necroptosis and myocardial injury. Hence, HNEAP may be a potential target for alleviating infarct size and improving cardiac function after ischemia/reperfusion (I/R) injury.

## 2 Results

### 2.1 Identification and Characterization of Myocardial Necroptosis‐Associated piRNAs

**Figure 1:**
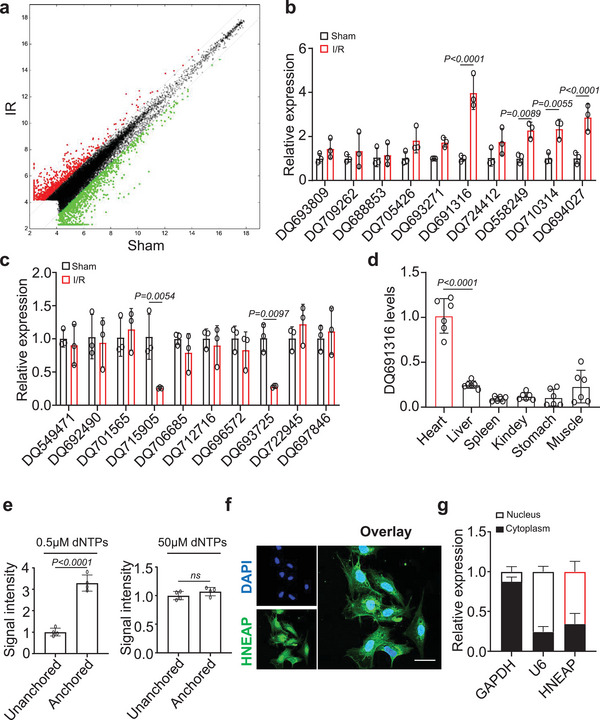
Identification of HNEAP in cardiomyocytes. a) The piRNA microarray data. b,c) Randomly detect the expression level of piRNAs up‐regulated (b) or down‐regulated (c) in I/R injured mouse hearts by qPCR (*n* = 3 independent experiments). d) Determination of relative expression of DQ691316 in different tissues of adult mice by qPCR (*n* = 6 independent experiments). e) Detection of 2′‐O‐methylation at the 3′ end of HNEAP using RTL‐P approach. RT‐PCR reaction was performed with an unanchored or anchored RT primer at different concentrations of dNTPs. f) Representative images of fluorescence in situ hybridization with junction‐specific probes of HNEAP demonstrate its subcellular localization. Green represents HNEAP and blue represents nuclei. Bar = 25 µm. g) The levels of HNEAP in the cytoplasm or nuclear fractions of cardiomyocytes were determined by qPCR. U6 and GAPDH were used as internal controls (*n* = 3 independent experiments). Data are presented as Mean ± SD. Two‐way ANOVA test (b,c) or one‐way ANOVA test (d,e).

piRNAs participate in the pathophysiological processes of many cardiovascular diseases, including myocardial hypertrophy and myocardial injury.^[^ ^19^ ^]^ Myocardial injury can lead to cardiomyocyte apoptosis and necroptosis.^[^ ^20^, ^21^ ^]^ To systematically identify and study the functions of piRNAs involved in cardiomyocyte necroptosis, we first performed a piRNA microarray analysis in I/R‐injured mouse hearts (**Figure** **1**a; Table S1, Supporting Information). We randomly selected 20 piRNAs (ten upregulated and ten downregulated) that were differentially expressed in the microarray results compared to those sham‐operated hearts and verified their expression levels by qRT‐PCR. We found that compared with the sham group, four piRNAs were significantly upregulated, and two were downregulated (Figure 1b,c). We also measured the levels of these piRNAs in cardiomyocytes treated with hypoxia/reoxygenation (H/R) (Figure S1a,b, Supporting Information). Among these, the level of piRNA DQ691316 in cardiomyocytes injured by H/R significantly increased. We next examined the levels of DQ691316 in various tissues and found that it was more abundant in the heart (Figure 1d). Compared to cardiac fibroblasts, DQ691316 was mainly expressed in cardiomyocytes (Figure S1c, Supporting Information). Based on these results, we hypothesized that DQ691316 may have a potential regulatory function in reperfusion‐induced cardiac injury and chose DQ691316 for further research. We named this unidentified piRNA heart‐necroptosis‐associated piRNA (HNEAP) and identified that HNEAP consists of 2′‐O‐methylation at the 3′‐end^[^ ^22^ ^]^ (Figure 1e). Subsequently, we observed that HNEAP was distributed in the nucleus and cytoplasm (Figure 1f,g).

### 2.2 Inhibition of HNEAP Blocks H/R‐Induced Cardiomyocyte Necroptosis

**Figure 2:**
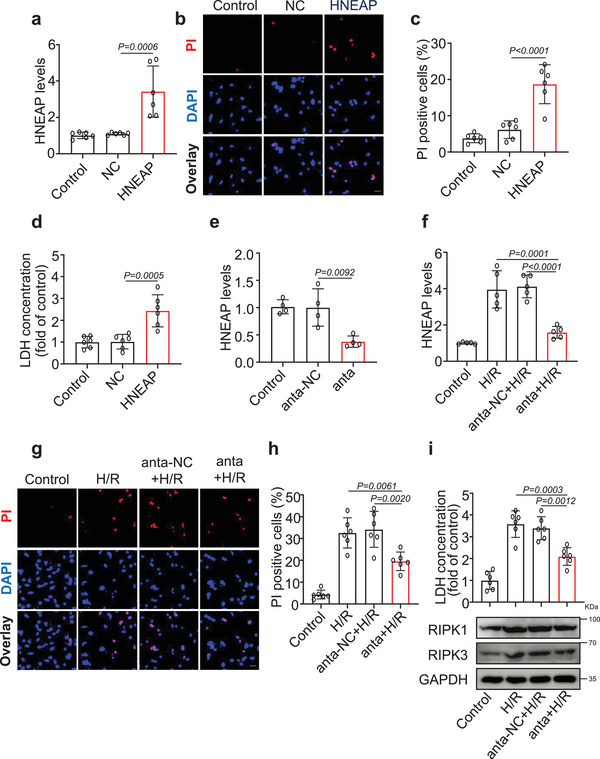
Knockdown of HNEAP attenuates H/R‐induced cardiomyocyte necroptosis. a–d) The isolated neonatal mice cardiomyocytes were transfected with HNEAP agomir (HNEAP) or its negative control (NC) for 24 h. a) The expression level of HNEAP was analyzed by qPCR (*n* = 6 independent experiments). b) Necroptosis was determined by the PI staining. DAPI indicates Nucleus. Bar = 25 µm. c) Quantitative analysis of the percentage of necroptotic cells (*n* = 6 independent experiments). d) The activity of lactate dehydrogenase (LDH) in cardiomyocytes after transfection with HNEAP or NC. (*n* = 6 independent experiments). e) The isolated neonatal mice cardiomyocytes were transfected with HNEAP antagomir (anta) or its negative control (anta‐NC) for 24 h. The expression level of HNEAP was analyzed by qPCR (*n* = 4 independent experiments). f–i) The isolated neonatal mice cardiomyocytes were transfected with HNEAP antagomir (anta) or its negative control (anta‐NC) for 24 h and then cells were treated with H/R as described in the Experimental Section. f) qPCR analysis of the expression level of HNEAP (*n* = 5 independent experiments). g) Necroptosis was determined by the PI staining. DAPI indicates Nucleus. Bar = 25 µm. h) Quantitative analysis of the percentage of necroptotic cells (*n* = 6 independent experiments). i) The upper panel shows the activity of LDH in cardiomyocytes after transfection with anta or anta‐NC and treated with H/R (*n* = 6 independent experiments). The lower panel is representative western blot showing the expression of RIPK1 and RIPK3. Data are presented as Mean ± SD. All data were analyzed using one‐way ANOVA.

To evaluate the function of HNEAP in cardiomyocyte necroptosis, we used H/R‐induced cardiomyocyte injury model. H/R induced cardiomyocyte necroptosis (Figure S1d,e, Supporting Information) and HNEAP expression (Figure S1f, Supporting Information) at different time points. Enforced expression of HNEAP (**Figure** **2**a) caused cardiomyocyte necroptosis, as indicated by an increased number of PI‐positive cardiomyocytes (Figure 2b,c) and increased LDH activity (Figure 2d). In contrast, knockdown of HNEAP using an inhibitor (Figure 2e,f) attenuated H/R‐induced cardiomyocyte necroptosis (Figure 2g–i). Furthermore, we examined the effect of HNEAP on cardiomyocyte apoptosis and observed that HNEAP had no effect on apoptosis in cardiomyocytes (Figure S2a,b, Supporting Information). Taken together, these results suggest that HNEAP participate in the regulation of cardiomyocyte necroptosis.

### 2.3 Knockout of HNEAP Attenuates Cardiac Damage and Necroptosis under I/R Injury

**Figure 3:**
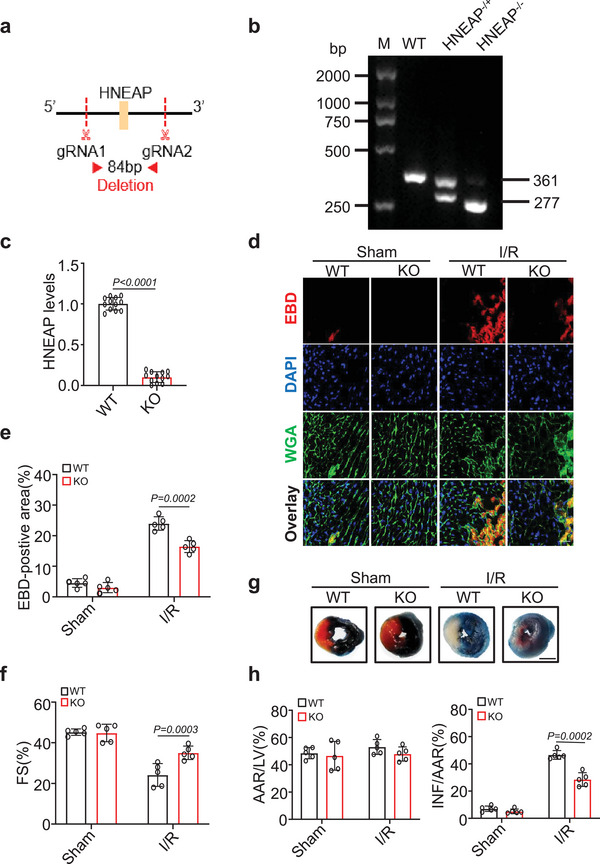
HNEAP deficiency ameliorates I/R‐induced necroptosis and myocardial injury. a) Schematic diagram of HNEAP location. CRISPR‐Cas9 gene editing system was used to knock out the genomic sequence of HNEAP and generation of the knockout mouse. b) Genotyping of HNEAP‐knockout (KO) mice by PCR and agarose electrophoresis. c) The expression level of HNEAP in wild‐type (WT) and HNEAP‐KO mouse hearts were analyzed by qPCR (*n* = 12 mice per group). d) WT and HNEAP‐KO mouse hearts were stained with Evans blue dye (EBD) to determine cell necroptosis. DAPI stands for nucleus. Immunostaining of wheat germ agglutinin (WGA) (green) labeled cardiomyocytes. EBD (red) marks necroptotic cardiomyocytes. Bar = 25 µm. e) Quantitative analysis of the percentage of necroptotic cardiomyocytes (*n* = 5 mice per group). f) Cardiac function (fractional shortening, FS%) of WT or HNEAP‐KO mouse hearts after I/R injury was measured by echocardiography (*n* = 5 mice per group). g) The representative photos of the midventricular myocardial slices stained with EBD‐TTC showed that the infarcted area of the heart in HNEAP‐KO mice decreased after I/R. Bar = 2 mm. h) Quantitative analysis of left ventricular infarct size. Area at risk (AAR), infarct area (INF) (*n* = 5 mice per group). Data are presented as Mean ± SD. Two‐sided Student's *t*‐test (c) or two‐way ANOVA test (e,f,h).

Cardiomyocyte necroptosis is an important factor in the pathophysiology of various cardiac diseases, including I/R injury.^[^ ^23^, ^24^ ^]^ To investigate whether HNEAP is functionally related to myocardial injury, we generated HNEAP‐knockout (HNEAP‐KO) mouse using CRISPR technology (**Figure** **3**a). Genotyping (Figure 3b) and qRT‐PCR analysis (Figure 3c) confirmed the deletion of HNEAP in KO mice. We did not observe significant morphological (Figure S2c,d, Supporting Information) or functional phenotypic alterations (Figure S2e, Supporting Information) in HNEAP‐KO hearts compared with wild type mouse hearts under basal conditions. To acquire a comprehensive understanding of the function of HNEAP in I/R injury, mouse hearts were ischemia for 1 h followed by reperfusion for 3 h (Figure S2f, Supporting Information). In contrast to sham‐treated hearts, Evans blue dye (EBD) can penetrate I/R‐injured heart sections, marking necroptotic cardiomyocytes red.^[^ ^25^ ^]^ Cardiomyocyte necroptosis was significantly reduced in the hearts of HNEAP‐KO mice during I/R injury (Figure 3d,e), and HNEAP‐KO mice showed improved ventricular function (Figure 3f) and reduced infarct size (Figure 3g,h) compared to WT mice with I/R injury. We then sought to determine the effect of HNEAP KO on cardiomyocyte apoptosis, and the results showed that the knockout of HNEAP had no inhibitory effect on I/R‐induced apoptosis (Figure S2g, Supporting Information). In summary, these data suggest that HNEAP deficiency attenuates cardiomyocyte necroptosis and alleviates cardiac dysfunction caused by I/R injury.

### 2.4 HNEAP Binds to DNMT1 and Regulates Its 5‐Methylcytosine Methylation Activity

**Figure 4:**
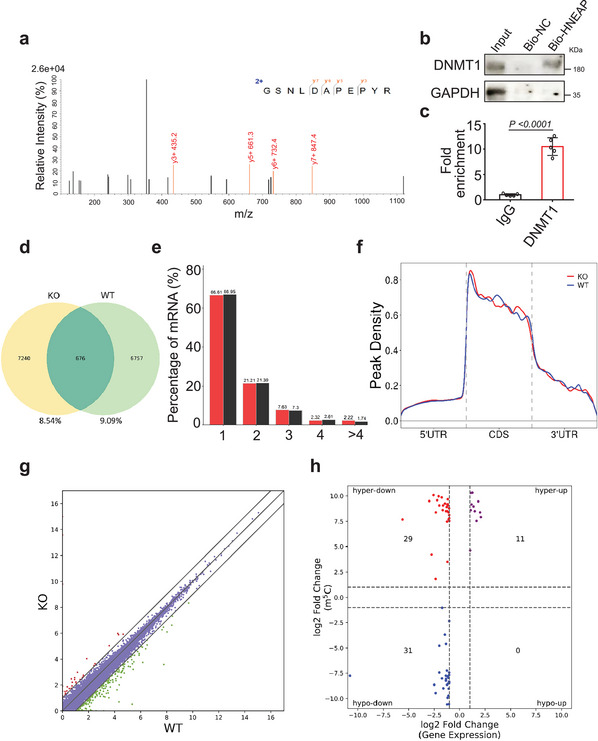
HNEAP binds to DNMT1 and regulates its 5‐methylcytosine methylation activity. a) Identification of HNEAP binding protein (detected DNMT1) using LC‐MS/MS. b) In cardiomyocytes, RNA pull‐down assay was carried out using biotinylated HNEAP (Bio‐HNEAP) or negative control (Bio‐NC) and western blot analysis showing HNEAP binds with DNMT1 protein. c) RNA immunoprecipitation using DNMT1 specific antibody followed by qPCR analysis showing HNEAP enriched in DNMT1 fraction (*n* = 5 independent experiments). d–h) m^5^C methylated RNA immunoprecipitation and sequencing (meRIP‐seq) was performed in KO and WT mouse hearts. d) Numbers of m^5^C peaks were detected in KO (left circle) and WT (right circle) mouse hearts. e) Percentage of mRNAs with different numbers of m^5^C peaks. f) Metagene profile showing the distribution of m^5^C peaks across the length of transcripts composed of three rescaled non‐overlapping segments 5′UTR, CDS, and 3′UTR in KO and WT mouse hearts. g) Scatter plot of differential expression of mRNAs assessed from RNA‐seq data. Red dots denote up‐regulated genes and green dots denote down‐regulated genes. h) Correlation between the level of gene expression (overall transcript) and changes in m^5^C level in KO mouse hearts compared to WT. Data are presented as Mean ± SD. Two‐sided Student's *t*‐test (c).

We explored the molecular mechanisms by which HNEAP regulates necroptosis in cardiomyocytes. Epigenetic modification of mRNA participates in many basic biological processes and is of great significance.^[^ ^26^, ^27^ ^]^ Our previous studies have shown that piRNAs regulate NAT10‐dependent ac^4^C acetylation.^[^ ^19^ ^]^ To further investigate the functional correlation between piRNA and mRNA epigenetic regulation, we performed RNA pull‐down assays using biotinylated HNEAP and detected epigenetically related molecules that interacted with HNEAP in cardiomyocytes (Figure S3a,b, Supporting Information). Proteins bound to HNEAP were identified using liquid chromatography‐tandem mass spectrometry (LC‐MS/MS). Among these proteins, we selected DNA methyltransferase 1 (DNMT1), whose function in necroptosis regulation is unknown (**Figure** **4**a). In vitro RNA pull‐down assay using biotinylated HNEAP, followed by immunoblotting, confirmed that HNEAP bound to DNMT1 (Figure 4b). After RIP, we conducted qPCR and observed that HNEAP was enriched in the DNMT1 group (Figure 4c), suggesting that HNEAP and DNMT1 interact with each other in vivo. In addition, we observed that the knockout or overexpression of HNEAP did not significantly alter the levels of DNMT1 mRNA and protein in cardiomyocytes (Figure S3c–f, Supporting Information). These results suggested that HNEAP interacts with DNMT1 and is involved in the regulation of 5‐methylcytosine methylation.

### 2.5 m^5^C Methylation in HNEAP‐KO Mouse Hearts

To examine the mechanism of HNEAP regulation of 5‐methylcytosine modification, we performed m^5^C methylated RNA immunoprecipitation sequencing (meRIP‐seq) in HNEAP‐KO and WT mouse hearts (Table S2, Supporting Information). Among all the detected m^5^C mRNA transcripts (Figure 4d), more than half contained one m^5^C peak (Figure 4e). In both WT and HNEAP‐KO mice, m^5^C peaks were predominantly found in coding sequences (CDSs) near start and stop codon**s** (Figure 4f). Next, we performed RNA‐seq analysis in HNEAP KO and WT mouse hearts to explore the relationship between m^5^C modification and gene expression (Figure 4g), and plotted m^5^C peak data against RNA‐seq data of gene expression to correlate the gene expression level with the m^5^C modification level (Figure 4h; Table S3, Supporting Information). We termed the upregulated peaks hypermethylated m^5^C peaks, including hyper‐down and hyper‐up genes. We termed the downregulated peaks hypomethylated m^5^C peaks, including hypo‐down and hypo‐up genes.

### 2.6 HNEAP Inhibits DNMT1‐Mediated m^5^C Modification and Promotes Atf7 mRNA Expression

**Figure 5:**
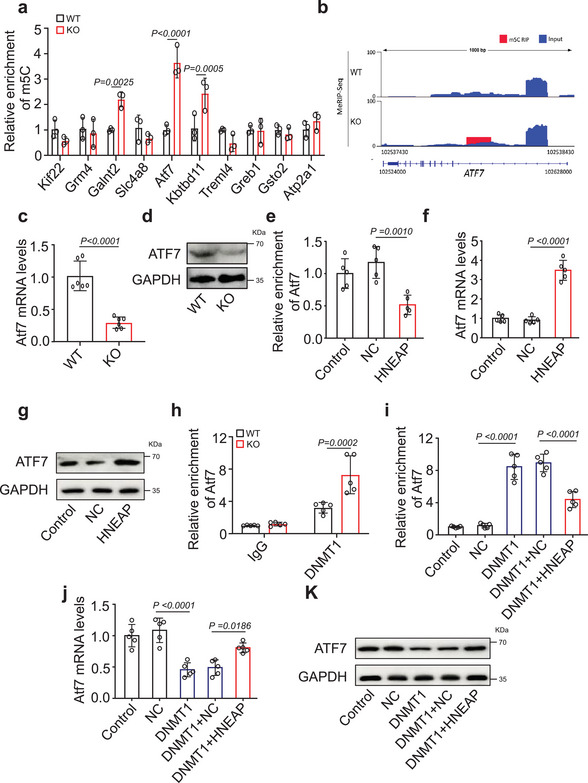
HNEAP inhibits m^5^C modification and promotes expression of Atf7 by targeting DNMT1. a) m^5^C methylated RNA immunoprecipitation and Quantitative real‐time PCR (acRIP‐qPCR) validation of m^5^C modification levels of genes selected from the results of meRIP‐seq and mRNA‐seq data in HNEAP‐KO and WT mouse hearts (*n* = 3 mice per group). b) Integrative Genomics Viewer (IGV) tracks displaying results of m^5^C‐seq read distribution in Atf7 mRNA of HNEAP‐KO and WT mouse hearts. c) The mRNA levels of Atf7 were detected by qPCR assay (*n* = 6 mice per group). d) The protein levels of Atf7 were detected by western blot assay. e) meRIP‐qPCR analysis in isolated cardiomyocytes treated with or without HNEAP agomir or NC shows the m^5^C modification level in Atf7 mRNA (*n* = 5 independent experiments). f) Expression levels of Atf7 mRNA in cardiomyocytes treated with HNEAP agomir or NC (*n* = 5 independent experiments). g) Expression levels of Atf7 protein in cardiomyocytes treated with HNEAP agomir or NC. h) RIP‐qPCR analysis in WT or HNEAP‐KO mouse hearts shows the level of Atf7 mRNA binding to DNMT1 (*n* = 5 mice per group). i) m^5^C enrichment level in Atf7 mRNA was detected in cardiomyocytes infected with adenovirus harboring DNMT1 or NC and transfected with HNEAP agomir or NC (*n* = 5 independent experiments). j) qPCR assay shows the mRNA level of Atf7 in cardiomyocytes infected with adenovirus harboring DNMT1 or NC and transfected with HNEAP agomir or NC (*n* = 5 independent experiments). k) Western blot assay shows the protein level of Atf7 in cardiomyocytes infected with adenovirus harboring DNMT1 or NC and transfected with HNEAP agomir or NC. Data are presented as Mean ± SD. Two‐way ANOVA test (a,h), two‐sided Student's *t*‐test (c), one‐way ANOVA test (e,f,i,j).

Next, we performed MeRIP‐qPCR and qPCR for differentially methylated and differentially expressed genes, respectively. Among these, the level of m^5^C modification of Atf7 in HNEAP‐KO hearts was significantly increased, and its expression level was dramatically reduced (**Figure** **5**a–d; Figure S4a–c, Supporting Information). In contrast, m^5^C enrichment at Atf7 decreased (Figure 5e), along with a significant increase in the levels of Atf7 mRNA and protein (Figure 5f,g) in HNEAP overexpressed cardiomyocytes. Based on these data, we selected Atf7 as the candidate target of m^5^C for the regulate of cardiomyocyte necroptosis. Next, we investigated how HNEAP upregulated Atf7 expression. In HNEAP‐KO mouse hearts, DNMT1 binding to Atf7 mRNA was significantly increased compared to that in WT mouse hearts (Figure 5h). In addition, forced expression of DNMT1 in cardiomyocytes increased the m^5^C modification level of Atf7 mRNA and decreased Atf7 mRNA and protein levels, and this effect was inhibited when HNEAP was overexpressed (Figure 5i–k). Taken together, these observations indicate that HNEAP mediates m^5^C modification of Atf7 mRNA through DNMT1 and that this methylation modification affects Atf7 mRNA expression.

### 2.7 Knockdown of Atf7 Attenuates Cardiomyocyte Necroptosis

**Figure 6:**
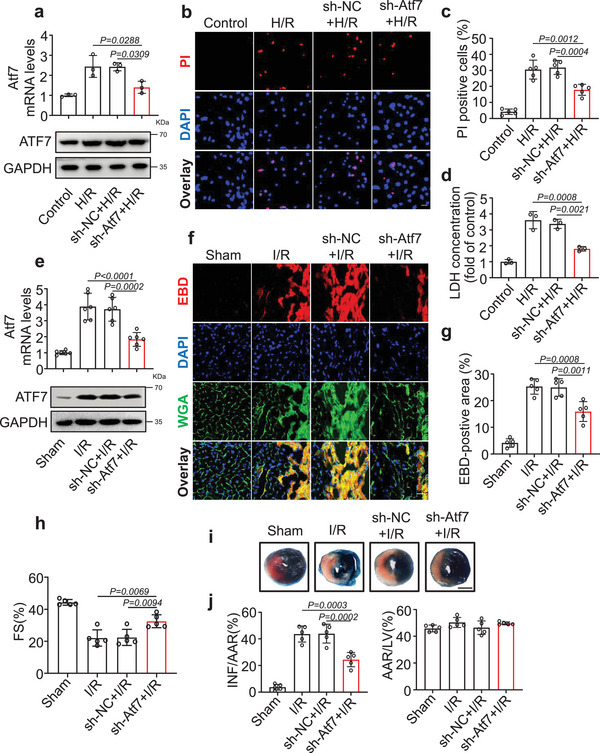
Inhibition of ATF7 attenuates cardiomyocyte necroptosis in vitro and in vivo. a–d) Isolated neonatal mice cardiomyocytes were infected with Atf7 shRNA (sh‐Atf7) or its negative control (sh‐NC) adenovirus for 24 h and then cells were treated with H/R. a) The upper panel shows the expression of Atf7 mRNA (*n* = 3 independent experiments). The lower panel is representative western blot showing the expression of ATF7. b) Necroptosis was determined by the PI staining. DAPI indicates Nucleus. Bar = 25 µm. c) Quantitative analysis of the percentage of necroptotic cells (*n* = 5 independent experiments). d) The activity of LDH in cardiomyocytes after transfection with sh‐Atf7 or sh‐NC and treated with H/R (*n* = 3 independent experiments). e–j) sh‐Atf7 or sh‐NC were injected into mice and I/R‐induced heart injury was performed 5 days after the injection. e) The mRNA (upper panel) and protein (lower panel) levels of Atf7 were detected by qPCR assay and western blot assay (*n* = 6 mice per group). f) Mouse hearts were stained with EBD to determine cell necroptosis. Bar = 25 µm. g) Quantitative analysis of the percentage of necroptotic cardiomyocytes (*n* = 5 mice per group). h) Cardiac function (FS%) of mouse hearts after I/R was measured by echocardiography (*n* = 5 mice per group). i) The representative photos of the midventricular myocardial slices stained with EBD‐TTC. Bar = 2 mm. j) Quantitative analysis of left ventricular infarct size. (*n* = 5 mice per group). Data are presented as Mean ± SD. All data were analyzed using one‐way ANOVA.

ATF7 is a vertebrate member of the ATF2 transcription factor subfamily that belongs to the ATF/CREB protein superfamily. These proteins are characterized by the presence of B‐Zip DNA‐binding domains.^[^ ^28^, ^29^, ^30^ ^]^ ATF/CREB play an important role in energy metabolism and cell growth; however, their function in cardiac tissues remains unclear.^[^ ^31^ ^]^ To confirm the function of ATF7 in the regulation of cardiomyocyte necroptosis, we knocked down the ATF7 in cardiomyocytes (Figure S5a, Supporting Information), and the results showed that knockdown of ATF7 alone had no effect on cardiomyocytes necroptosis (Figure S5b,c, Supporting Information). However, Atf7 knockdown blocked H/R‐induced cardiomyocyte necroptosis (**Figure** **6**a–d; Figure S5d, Supporting Information). In vivo, Atf7 mRNA and protein levels remarkably increased after I/R surgery (Figure 6e). Atf7 knockdown (Figure 6e) reduced cardiomyocyte necroptosis (Figure 6f,g), ameliorated ventricular function (Figure 6h), and reduced infarct size (Figure 6i,j). In additon, we found that ATF7 knockdown had no effect on H/R‐induced cardiomyocyte apoptosis (Figure S5e,f, Supporting Information). We further explored the effects of HNEAP on ATF7 expression. HNEAP knockdown decreased ATF7 expression level (Figure S6a, Supporting Information); it also inhibited H/R‐induced ATF7 expression (Figure S6b, Supporting Information) and necroptosis; these effects were attenuated by DNMT1 silencing (Figure S6c–e, Supporting Information). Taken together, these data indicate that Atf7 plays a critical role in the regulation of cardiomyocyte necroptosis, and Atf7 is a direct downstream target of HNEAP and DNMT1.

### 2.8 Atf7 Modulates the Expression of CHMP2A in Cardiomyocytes Necroptosis

**Figure 7:**
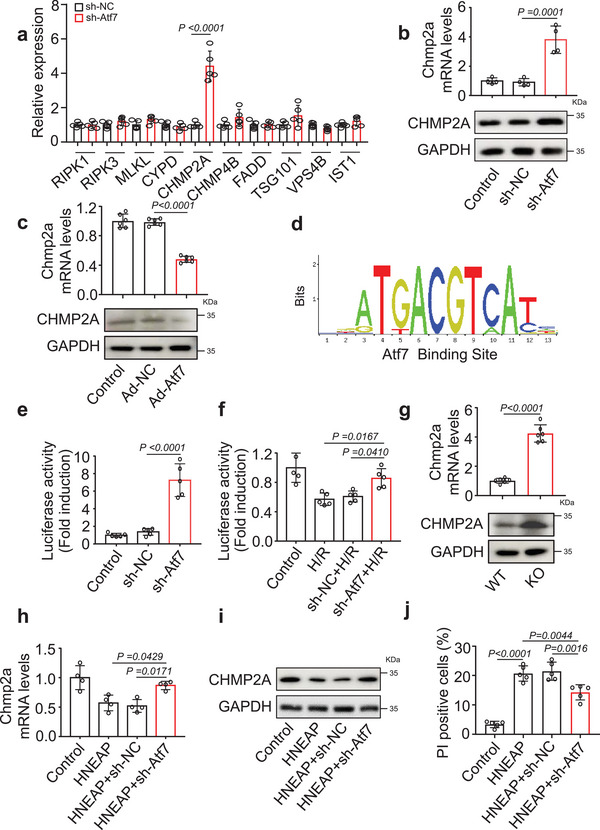
Atf7 regulates CHMP2A expression during cardiomyocyte necroptosis. a) Cardiomyocytes were infected with shRNA specifically silencing of Atf7 (sh‐ATF7) or its negative control (sh‐NC) and expression levels of key genes involved in cardiomyocyte necroptosis were determined by qPCR (*n* = 5 independent experiments). b) Cardiomyocytes were infected with sh‐Atf7 or sh‐NC and Chmp2a mRNA was assessed by qPCR (upper) (*n* = 4 independent experiments). The expression levels of Chmp2a protein assessed by western blot (lower). c) Cardiomyocytes were infected with adenoviral vector harboring Atf7 (Ad‐Atf7) or its negative control (Ad‐NC) and Chmp2a mRNA was assessed by qPCR (upper) (*n* = 6 independent experiments). The expression levels of Chmp2a protein assessed by western blot (lower). d) Motif analysis of the Atf7 bound regions. e,f) Luciferase activity was carried out (*n* = 4–5 independent experiments). g) The mRNA levels of Chmp2a were detected by qPCR (upper) (*n* = 6 mice per group). The protein levels of Chmp2a were detected by western blot (lower). h–j) Cardiomyocytes were infected with sh‐Atf7 or sh‐NC and transfected with HNEAP for 24 h. h) The mRNA levels of Chmp2a were detected by qPCR assay (n = 4 independent experiments). i) The protein levels of Chmp2a were detected by western blot assay. j) Quantitative analysis of the percentage of necroptotic cells (*n* = 5 independent experiments). Data are presented as Mean ± SD. Two‐way ANOVA test (a), one‐way ANOVA test (b,c,e,f,h,j), two‐sided Student's *t*‐test (g).

To investigate the downstream effectors of Atf7, we evaluated the expression levels of critical factors associated with necroptosis, as reported in previous studies. Specifically, the expression level of the key necroptosis molecule, CHMP2A, increased upon Atf7 silencing in cardiomyocytes (**Figure** **7**a,b). Conversely, Atf7 overexpression decreased the mRNA and protein levels of Chmp2a (Figure 7c), indicating an inverse correlation between Atf7 and Chmp2a. Next, we evaluated whether Atf7 directly targets Chmp2a. We constructed a Chmp2a promoter containing an Atf7 binding site (Figure 7d) and tested the effect of Atf7 on its activity using a luciferase reporter assay. Atf7 knockdown increased luciferase activity of the Chmp2a construct (Figure 7e). The H/R‐induced decrease in the transcription of Chmp2a was attenuated upon Atf7 knockdown in cardiomyocytes (Figure 7f). These results suggest that Atf7 inhibits CHMP2A expression by directly binding to its promoter region of Chmp2a.

Next, we investigated the effects of CHMP2A on cardiomyocyte necroptosis. Chmp2a knockdown (Figure S7a, Supporting Information) promoted necroptosis (Figure S7b,c, Supporting Information). Furthermore, we investigated whether HNEAP regulated cardiomyocyte necroptosis by targeting CHMP2A. The results showed that Chmp2a mRNA and protein expression levels significantly increased in HNEAP‐KO mouse hearts compared to those in WT hearts (Figure 7g). HNEAP reduced Chmp2a expression level and induced necroptosis in cardiomyocytes, and these effects were reversed by Atf7 knockdown (Figure 7h–j, Figure S7d,e, Supporting Information). Next, we examined whether HNEAP mediates cardiomyocyte necroptosis through targeting Atf7 and Chmp2a. The results showed that knockdown of HNEAP inhibited H/R‐induced cardiomyocyte necroptosis, and this effect was attenuated when Chmp2a was knocked down (Figure S7f, Supporting Information). These data suggest that Atf7 and CHMP2A are direct downstream molecules of HNEAP that regulate cardiomyocyte necroptosis.

## 3 Discussion

Herein, we found that HNEAP is a necroptosis‐associated piRNA that accelerates cardiomyocyte necroptosis by regulating RNA 5‐methylcytosine (m^5^C) modification. HNEAP inhibited Atf7 mRNA methylation by recruiting DNMT1. This results in the activation of Atf7 mRNA transcription and increased expression level of the transcription factor Atf7, which, in turn, inhibits the transcription of CHMP2A, an anti‐necroptotic factor (Figure S8, Supporting Information). In conclusion, our results indicate that HNEAP is a critical pro‐necroptotic factor in the epigenetic modification of Atf7 mRNA.

Previous studies have identified abundant differentially expressed piRNAs in injured hearts and cardiac tissues with pathological hypertrophy. Currently, only a few piRNAs have been functionally characterized, and the functions of most piRNAs in physiological and pathological processes in the heart remain unclear. Our results showed that the expression level of HNEAP in heart tissue and cardiomyocytes was higher than that in other tissues and cardiac fibroblasts, demonstrating that HNEAP has heart‐specific functions. Epigenetics is the study of heritable modifications involved in gene expression and regulation.^[^ ^32^ ^]^ DNA methylation plays a critical role in the regulation of gene activity. DNMTs can catalyze the addition of methylation markers to genomic DNA, which is indispensable for methylation maintenance.^[^ ^33^ ^]^ There are three common types of DNMTs, namely DNMT1, DNMT3A, and DNMT3B. Among them, DNMT1 is the main maintenance methylase, while DNMT3A and DNMT3B are responsible for de novo methylation.^[^ ^34^, ^35^ ^]^ However, DNMT1 is also involved in de novo methylation.^[^ ^36^ ^]^ In general, decreased level of DNA methylation leads to genomic instability, whereas aberrant site‐specific methylation of gene promoters leads to specific gene silencing.^[^ ^37^ ^]^ Many DNA‐binding chromatin‐modifying enzymes can also bind to RNA, and this binding is thought to play a role in the recruitment and regulation of enzyme activity.^[^ ^35^ ^]^ However, the detailed mechanisms underlying RNA‐mediated regulation require further exploration. DNMT1 can directly associate with multiple RNAs, and different RNAs have distinct effects on the activity and localization of DNMT1. The long non‐coding RNA (lncRNA) Dacor1 interacts with DNMT1, leading to abnormal global DNA methylation.^[^ ^38^ ^]^ In addition, some miRNAs interact directly with DNMT1. The binding of miR‐155‐5p to DNMT1 can inhibit its activity of DNMT1 enzyme, leading to hypomethylation in some regions; this abnormal methylation state can cause changes in gene expression levels.^[^ ^39^ ^]^ Here, we demonstrated that HNEAP directly binds to the RNA methyltransferase DNMT1 but does not affect its expression level. However, the specific binding mode of HNEAP remains unclear. Whether HNEAP directly binds to the catalytic domain of DNMT1 or alters its activity requires further investigation. Other factors may be involved in the binding of HNEAP to DNMT1. Based on previous findings that piRNAs depend on PIWI proteins to regulate gene expression, we speculated that HNEAP and PIWI proteins, including PIWIL4 and PIWIL2, may form a complex with DNMT1 and affect its RNA methylation function. In future studies, we will further explore whether PIWIL4 or PIWIL2 can interact with HNEAP to form a complex that participates in the regulation of cardiomyocyte necroptosis. Furthermore, interactions between piRNAs and DNMTs may not be limited to specific methyltransferases. It remains unknown whether HNEAP interacts with other DNMTs, such as DNMT3A or DNMT3B.

In the present study, we identified Atf7 mRNA as a downstream m^5^C methylation target in the HNEAP–DNMT1 complex. We observed that the overexpression of HNEAP reduced the m^5^C methylation of Atf7 mRNA and increased its mRNA and protein levels. Therefore, we hypothesized that m^5^C methylation modifies Atf7 mRNA to reduce the expression level of Atf7 by regulating its stability. Atf7 belongs to the ATF/cAMP response element binding (CREB) protein superfamily and contains a B‐Zip DNA binding domain. ATF/CREB plays an important role in energy metabolism and cell growth^[^ ^31^ ^]^ and participates in regulating the development of the heart and somatic cells.^[^ ^40^ ^]^ Our results showed that Atf7 knockdown reduced cardiomyocyte necroptosis induced by pathological stimuli, suggesting that ATF7 functions as a pro‐necroptotic transcription factor during heart disease development.

The present study demonstrated that ATF7 can reverse‐regulate CHMP2A transcription during H/R‐induced cardiomyocyte necroptosis and I/R‐induced myocardial injury. CHMP2A belongs to the chromatin‐modifying protein/charged multivesicular body protein (CHMP) family.^[^ ^41^ ^]^ These proteins are components of the endosomal sorting complex required for transport‐III (ESCRT‐III), a complex involved in the degradation of surface receptor proteins and formation of endocytic multivesicular bodies (MVBs).^[^ ^42^, ^43^ ^]^ Some CHMPs have both nuclear and cytoplasmic/vesicular distribution. CHMP1A is necessary for MVB formation and cell cycle regulation.^[^ ^44^ ^]^ Silencing of CHMP2A can lead to spontaneous necrosis.^[^ ^45^ ^]^ Consistent with these studies, our results suggest that CHMP2A is involved in the regulation of cardiomyocyte necroptosis. Additionally, we demonstrated that CHMP2A, a transcriptional target of ATF7, participates in the regulation of cardiac injury. Therefore, our study highlights the critical role of mRNA m^5^C modification in the regulation of cardiomyocyte necroptosis and provides novel understanding of the mechanisms of piRNA‐mediated post‐transcriptional gene regulation. Our results showed that, in addition to mRNA m^6^A methylation and ac^4^C acetylation, mRNA m^5^C methylation modification also participates in the pathological process of cardiac disease. Furthermore, the marked improvement in cardiac function and significant reduction in infarct size in HNEAP‐deficient mouse hearts suggest that targeting HNEAP may be an effective therapeutic strategy for alleviating ischemic injury in the heart.

## 4 Experimental Section

An expanded Experimental Section is supplied in the supplementary data file.

### Cell Isolation and Culture

The hearts of newborn mice (1–2 days of age) were washed with cold PBS 3–4 times. Then the ventricular regions were separated and chopped in HEPES‐buffered saline. The tissue was dispersed in the digestive solution containing pancreatin (Sigma, St. Louis, Missouri, USA) and collagenaseII (Worthington, Lakewood, USA) at 37 °C, and was treated by a series of repeated digestion. The collected cells were resuspended in Dulbecco's modified Eagle's medium/F‐12 (DMEM/F‐12) (Servicebio, G4610, Wuhan, China) containing 5% Fetal bovine serum (ExCell, Shanghai, China) and 1% Penicillin‐Streptomycin Liquid (Solarbio, Beijing, China). After 1.5 h pre‐plated at 37 °C differential adherent isolation and bromodeoxyuridine (BrdU) (Sigma, St. Louis, Missouri, USA) to the purification of cardiomyocytes. Then cardiomyocytes were treated with hypoxia‐reoxygenation (H/R) for the indicated periods. In brief, the cardiomyocytes were pretreated in DMEM/F‐12 (without Glucose) (Meilunbio, MA0598, Dalian, China) medium for 12 h and then placed in a hypoxic environment at 37 °C. After completion, DMEM/F12 medium containing 5% serum was replaced, and the cells were reoxygenated in an incubator with 5% CO_2_ and 95% O_2_ at 37°C for 6 h. Then collected cells for subsequent experiments.

### Animal Experiments and Generation of HNEAP‐Knockout (HNEAP‐KO) Mice

HNEAP‐knockout mice were generated by using clustered regularly interspaced short palindromic repeats associated protein 9 (CRISPR/Cas9) gene‐editing system (Cyagen Biosciences Inc. Guangzhou, China). HNEAP^+/−^ mice were interbred to generate KO mice (HNEAP^−/−^), which were used for further studies. Mice were genotyped by PCR with forward primer: 5′‐CCAGAACATCTGACAGATTTTAAGTCAAG‐3′ and reverse primer: 5′‐TTATCTGGAGACAGGGAGTGTTTG‐3′. The PCR products were further PCR screened to verify that the HNEAP was correctly deleted. All experiments were performed on HNEAP^−/−^ mice and their WT littermates.

All mice were kept in a temperature‐controlled animal facility under a normal light/dark cycle with free access to food and water for the whole experiment. All procedures with animals were approved by the Ethics Committee of Animal Experiments of the Qingdao University and performed in accordance with the guidelines from Directive 2010/63/EU of the European Parliament. Mice were anaesthetised with isoflurane in oxygen (4% induction, 1–1.5% maintenance). At the end of the study, all animals were euthanized in a CO_2_ chamber.

### Myocardial Ischemia‐Reperfusion (I/R) Injury

Adult male mice were aged 8–10 weeks were selected for the experiment. After anesthesia, connect the trachea to assist breathing, the left chest was carefully opened to expose the heart, and ligate the left anterior descending (LAD) coronary artery in the temporal region. Ischemic treatment was performed for 60 min. After ischemia treatment for 60 min, open the tight ferrule and reperfusion for 3 h to complete the operation. For sham‐operated mice, the same procedure was carried out except for occlusion of the LAD. During the whole procedure, the mice were placed on a 37 °C heating pad in a supine position.

For Atf7 knockdown or overexpression in vivo, the Atf7 knockdown or overexpression vector was constructed by adenovirus (Hanbio, Shanghai, China). Mice were given tail vein injection 5 days before Sham or I/R operation (5 × 10^11^vg/mouse).

### Echocardiography Measurement

After the whole operation, echocardiographic measurements were performed on mice using the Vevo2100 imaging system (Visual Sonic) to evaluate cardiac function. Fractional shortening (FS) was calculated by this system.

### RNA Isolation and qRT‐PCR

Total RNA was isolated from cardiomyocytes and tissue samples with RNA isolater Total RNA Extraction Reagent (Vazyme, R401‐01, Nanjing, China), and reverse transcripted with Evo M‐MLV RT Kit with gDNA Clean for qPCR II (Accurate Biotechnology (Hunan), AG11711, Changsha, China) according to the instructions. Then a MonAmpTM ChemoHS qPCR Mix (Monad, MQ00401, China) in a Quantstudio5 qPCR instrument (Thermo Fisher, Singapore) was used for qRT‐PCR. The results were normalized against U6 or GAPDH. For qRT‐PCR analysis, the primer sequences for specific genes are provided in Table S4 (Supporting Informtion).

### Protein Isolation and Western Blot

Total proteins were isolated from cardiomyocytes and tissue samples with RIPA lysate (Solarbio, R0020, Beijing, China) containing protease inhibitor (Meilunbio, MA0001, Dalian, China) at low temperature. The total protein was separated on SDS‐PAGE and transferred to PVDF membranes. After blocking with 5% BSA for 2 h at room temperature, the antibody was used for incubation. Finally, western blot analysis was performed using ECL chemiluminescence. Antibodies used for Western blots included anti‐5‐methylcytosine (Abcam, ab10805, USA), RIPK1 (ABclonal, A7414, 1:1000 dilution), RIPK3 (Affinity biosciences, DF10141, 1:1000 dilution), anti‐ATF7 (Zen‐Bioscience, 381411, 1:500 dilution), anti‐DNMT1 (ABclonal, A16833, 1:800 dilution), anti‐CHMP2A (Proteintech, 10477‐1‐AP, 1:800 dilution), anti‐cTnT (Bioss, bs‐10648R, 1:800 dilution), anti‐GAPDH (Zen‐Bioscience, 200306–7E4, 1:5000 dilution), goat anti‐Rabbit IgG (H+L) HRP (Affinity biosciences, S0001, 1:10 000 dilution), goat anti‐Mouse IgG (H+L) HRP (Affinity biosciences, S0002, 1:10 000 dilution) for WB.

### Statistical Analysis

All data were expressed as the mean ± SD. GraphPad Prism (GraphPad Software Inc., San Diego, CA) was used for statistical analysis. Student's *t*‐test was used for comparison between the two groups, and unpaired two‐sided test was used for all tests. One‐way analysis of variance (ANOVA) with Tukey's multiple comparisons test or two‐way ANOVA with Bonferroni's multiple comparisons test was used for multi‐group comparisons. The experiments were repeated at least three times independently, and similar results were obtained. When *p* < 0.05, it was considered to be statistically significant.

## Conflict of Interest

The authors declare no conflict of Interest.

## Author Contributions

K.W., F.‐H.L., L.‐Y.Z., X.‐M.Z., and X.‐Q.G. contributed equally to this work. K.W., J.T., and F.G. designed research. K.W., F.L., L.Z., X.Z., X.G., C.L., X.L., X.C., Y.Z., X.C., R.W., and R.L. performed experiments. K.W., F.L., L.Z., X.Z., and Y.Z. analyzed the data. K.W., K.W., J.T. and F.G. wrote the manuscript.

## Supporting information

Supporting Information

Supporting Information

Supporting Information

Supporting Information

Supporting Information

## Data Availability

The data that support the findings of this study are available from the corresponding author upon reasonable request.

## References

[1] Z. Zhao , X. Li , C. Gao , D. Jian , P. Hao , L. Rao , M. Li , Sci. Rep. 2017, 7, 39918. 28045102 10.1038/srep39918 PMC5206672

[2] T.‐R. Zhang , W.‐Q. Huang , Microvasc. Res. 2020, 129, 103983. 31953183 10.1016/j.mvr.2020.103983

[3] S. Cadenas , Free Radical Biol. Med. 2018, 117, 76. 29373843 10.1016/j.freeradbiomed.2018.01.024

[4] E. T. Chouchani , V. R. Pell , E. Gaude , D. Aksentijevic , S. Y. Sundier , E. L. Robb , A. Logan , S. M. Nadtochiy , E. N. J. Ord , A. C. Smith , F. Eyassu , R. Shirley , C.‐H. Hu , A. J. Dare , A. M. James , S. Rogatti , R. C. Hartley , S. Eaton , A. S. H. Costa , P. S. Brookes , S. M. Davidson , M. R. Duchen , K. Saeb‐Parsy , M. J. Shattock , A. J. Robinson , L. M. Work , C. Frezza , T. Krieg , M. P. Murphy , Nature 2014, 515, 431. 25383517 10.1038/nature13909 PMC4255242

[5] P. Vandenabeele , L. Galluzzi , T. Vanden Berghe , G. Kroemer , Nat. Rev. Mol. Cell Biol. 2010, 11, 700. 20823910 10.1038/nrm2970

[6] S. '. Zhou , W. Zhang , G. Cai , Y. Ding , C. Wei , S. Li , Y. Yang , J. Qin , D. Liu , H. Zhang , X. Shao , J. Wang , H. Wang , W. Yang , H. Wang , S. Chen , P. Hu , L. Sun , Cell Res. 2020, 30, 1063. 32839552 10.1038/s41422-020-00393-6 PMC7784988

[7] A. Kaczmarek , P. Vandenabeele , D. V. Krysko , Immunity 2013, 38, 209. 23438821 10.1016/j.immuni.2013.02.003

[8] H. Wang , L. Sun , L. Su , J. Rizo , L. Liu , L.‐F. Wang , F.‐S. Wang , X. Wang , Mol. Cell 2014, 54, 133. 24703947 10.1016/j.molcel.2014.03.003

[9] X.‐Q. Gao , C.‐Y. Liu , Y.‐H. Zhang , Y.‐H. Wang , L.‐Y. Zhou , X.‐M. Li , K. Wang , X.‐Z. Chen , T. Wang , J. Ju , F. Wang , S.‐C. Wang , Y. Wang , Z.‐Y. Chen , K. Wang , Cell Death Differ. 2022, 29, 527. 34588633 10.1038/s41418-021-00872-2 PMC8901615

[10] Q. Xu , S. Jitkaew , S. Choksi , C. Kadigamuwa , J. Qu , M. Choe , J. Jang , C. Liu , Z.‐G. Liu , Nat. Commun. 2017, 8, 425. 28871172 10.1038/s41467-017-00496-6 PMC5583178

[11] P. A. Dionisio , J. D. Amaral , C. M. P. Rodrigues , Int. Rev. Cell. Mol. Biol. 2020, 353, 31. 32381178 10.1016/bs.ircmb.2019.12.006

[12] H. Tu , Y.‐J. Zhou , L.‐J. Tang , X.‐M. Xiong , X.‐J. Zhang , Md S. Ali Sheikh , J.‐J. Zhang , X.‐J. Luo , C. Yuan , J. Peng , Eur. J. Pharmacol. 2021, 898, 173999. 33675785 10.1016/j.ejphar.2021.173999

[13] C. Schulte , T. Barwari , A. Joshi , T. Zeller , M. Mayr , Trends Mol. Med. 2020, 26, 583. 32470385 10.1016/j.molmed.2020.02.001

[14] T. Huang , W. Chen , J. Liu , N. Gu , R. Zhang , Nat. Struct. Mol. Biol. 2019, 26, 380. 31061524 10.1038/s41594-019-0218-x

[15] X. Yang , Y. Yang , B.‐F. Sun , Y.‐S. Chen , J.‐W. Xu , W.‐Y. Lai , A. Li , X. Wang , D. P. Bhattarai , W. Xiao , H.‐Y. Sun , Q. Zhu , H.‐L. Ma , S. Adhikari , M. Sun , Y.‐J. Hao , B. Zhang , C.‐M. Huang , N. Huang , G.‐B. Jiang , Y.‐L. Zhao , H.‐L. Wang , Y.‐P. Sun , Y.‐G. Yang , Cell Res. 2017, 27, 606. 28418038 10.1038/cr.2017.55 PMC5594206

[16] X. Zhang , Z. Liu , J. Yi , H. Tang , J. Xing , M. Yu , T. Tong , Y. Shang , M. Gorospe , W. Wang , Nat. Commun. 2012, 3, 712. 22395603 10.1038/ncomms1692 PMC3509206

[17] K. Bohnsack , C. Höbartner , M. Bohnsack , Genes 2019, 10, 102. 30704115 10.3390/genes10020102 PMC6409601

[18] X.‐Z. Li , Y‐.T Meng , J. Cell. Mol. Med. 2021, 25, 1383. 33400376 10.1111/jcmm.16221 PMC7875931

[19] K. Wang , L.‐Y. Zhou , F. Liu , L. Lin , J. Ju , P.‐C. Tian , C.‐Y. Liu , X.‐M. Li , X.‐Z. Chen , T. Wang , F. Wang , S.‐C. Wang , J. Zhang , Y.‐H. Zhang , J.‐W. Tian , K. Wang , Adv. Sci. 2022, 9, e2106058. 10.1002/advs.202106058 PMC8922123 35138696

[20] J. Pu , A. Yuan , P. Shan , E. Gao , X. Wang , Y. Wang , W. B. Lau , W. Koch , X.‐L. Ma , B. He , Eur. Heart J. 2013, 34, 1834. 22307460 10.1093/eurheartj/ehs011 PMC3689100

[21] H. Yin , X. Guo , Y. Chen , Y. Zeng , X. Mo , S. Hong , H. He , J. Li , R. Steinmetz , Q. Liu , J. Clin. Invest. 2022, 132, e152297. 34990405 10.1172/JCI152297 PMC8843707

[22] Z.‐W. Dong , P. Shao , L.‐T. Diao , H. Zhou , C.‐H. Yu , L.‐H. Qu , Nucleic Acids Res. 2012, 40, e157. 22833606 10.1093/nar/gks698 PMC3488209

[23] Z. Song , H. Song , D. Liu , B. Yan , D. Wang , Y. Zhang , X. Zhao , X. Tian , C. Yan , Y. Han , Theranostics 2022, 12, 1267. 35154486 10.7150/thno.65716 PMC8771548

[24] X. Zhai , W. Wang , S. Sun , Y. Han , J. Li , S. Cao , R. Li , T. Xu , Q. Yuan , J. Wang , S. Wei , Y. Chen , Front. Cell Dev. Biol. 9, 721795. 10.3389/fcell.2021.721795 PMC8517475 34660582

[25] G. J. Lee , L. Yan , D. E. Vatner , S. F. Vatner , Basic Res. Cardiol. 2015, 110, 7. 25600225 10.1007/s00395-015-0461-1 PMC4357177

[26] W. Wang , G. Lu , H.‐B. Liu , Z. Xiong , H.‐D. Leung , R. Cao , A. L.‐Y. Pang , X. Su , P. W. N. Law , Z. Zhao , Z.‐J. Chen , W.‐Y. Chan , Adv. Sci. 2021, 8, e2100849. 10.1002/advs.202100849 PMC8425920 34247447

[27] Y. Wu , S. Zhan , Y. Xu , X. Gao , Life Sci. 2021, 278, 119565. 33965380 10.1016/j.lfs.2021.119565

[28] M. Gaire , B. Chatton , C. Kedinger , Nucleic Acids Res. 1990, 18, 3467. 1694576 10.1093/nar/18.12.3467 PMC330998

[29] T. W. Hai , F. Liu , W. J. Coukos , M. R. Green , Genes Dev. 1989, 3, 2083. 2516827 10.1101/gad.3.12b.2083

[30] T. Maekawa , H. Sakura , C. Kanei‐Ishii , T. Sudo , T. Yoshimura , J. Fujisawa , M. Yoshida , S. Ishii , EMBO J. 1989, 8, 2023. 2529117 10.1002/j.1460-2075.1989.tb03610.x PMC401081

[31] S. P. Persengiev , Apoptosis 2003, 8, 225. 12766482 10.1023/a:1023633704132

[32] A. Nebbioso , F. P. Tambaro , C. Dell'aversana , L. Altucci , PLoS Genet. 2018, 14, e1007362. 29879107 10.1371/journal.pgen.1007362 PMC5991666

[33] T. Liu , J. Wang , L. Sun , M. Li , X. He , J. Jiang , Q. Zhou , Cell Cycle 2021, 20, 1603. 34313525 10.1080/15384101.2021.1956090 PMC8409782

[34] L. Gao , M. Emperle , Y. Guo , S. A. Grimm , W. Ren , S. Adam , H. Uryu , Z.‐M. Zhang , D. Chen , J. Yin , M. Dukatz , H. Anteneh , R. Z. Jurkowska , J. Lu , Y. Wang , P. Bashtrykov , P. A. Wade , G. G. Wang , A. Jeltsch , J. Song , Nat. Commun. 2020, 11, 3355. 32620778 10.1038/s41467-020-17109-4 PMC7335073

[35] L. I. Jansson‐Fritzberg , C. I. Sousa , M. J. Smallegan , J. J. Song , A. R. Gooding , V. Kasinath , J. L. Rinn , T. R. Cech , RNA 2023, 29, 346. 36574982 10.1261/rna.079479.122 PMC9945446

[36] Y. Li , Z. Zhang , J. Chen , W. Liu , W. Lai , B. Liu , X. Li , L. Liu , S. Xu , Q. Dong , M. Wang , X. Duan , J. Tan , Y. Zheng , P. Zhang , G. Fan , J. Wong , G.‐L. Xu , Z. Wang , H. Wang , S. Gao , B. Zhu , Nature 2018, 564, 136. 30487604 10.1038/s41586-018-0751-5

[37] C. L. Esposito , I. Autiero , A. Sandomenico , H. Li , M. A. Bassal , M. L. Ibba , D. Wang , L. Rinaldi , S. Ummarino , G. Gaggi , M. Borchiellini , P. Swiderski , M. Ruvo , S. Catuogno , A. K. Ebralidze , M. Kortylewski , V. De Franciscis , A. Di Ruscio , Nat. Commun. 2023, 14, 99. 36609400 10.1038/s41467-022-35222-4 PMC9823104

[38] C. R. Merry , M. E. Forrest , J. N. Sabers , L. Beard , X.‐H. Gao , M. Hatzoglou , M. W. Jackson , Z. Wang , S. D. Markowitz , A. M. Khalil , Hum. Mol. Genet. 2015, 24, 6240. 26307088 10.1093/hmg/ddv343 PMC4599679

[39] G. Zhang , P.‐O. Estève , H. . G. Chin , J. Terragni , N. Dai , I. R. Corrêa , S. Pradhan , Nucleic Acids Res. 2015, 43, 6112. 25990724 10.1093/nar/gkv518 PMC4499142

[40] W. Zhou , L. Lin , A. Majumdar , X. Li , X. Zhang , W. Liu , L. Etheridge , Y. Shi , J. Martin , W. Van De Ven , V. Kaartinen , A. Wynshaw‐Boris , A. P. Mcmahon , M. G. Rosenfeld , S. M. Evans , Nat. Genet. 2007, 39, 1225. 17767158 10.1038/ng2112 PMC5578467

[41] S. Lata , G. Schoehn , J. Solomons , R. Pires , H. G. Göttlinger , W. Weissenhorn , Biochem. Soc. Trans. 2009, 37, 156. 19143622 10.1042/BST0370156

[42] Y. Takahashi , H. He , Z. Tang , T. Hattori , Y. Liu , M. M. Young , J. M. Serfass , L. Chen , M. Gebru , C. Chen , C. A. Wills , J. M. Atkinson , H. Chen , T. Abraham , H.‐G. Wang , Nat. Commun. 2018, 9, 2855. 30030437 10.1038/s41467-018-05254-w PMC6054611

[43] Y. Zhen , H. Spangenberg , M. J. Munson , A. Brech , K. O. Schink , K.‐W. Tan , V. Sørensen , E. M. Wenzel , M. Radulovic , N. Engedal , A. Simonsen , C. Raiborg , H. Stenmark , Autophagy 2020, 16, 826. 31366282 10.1080/15548627.2019.1639301 PMC7158923

[44] H. T. H. Tsang , J. W. Connell , S. E. Brown , A. Thompson , E. Reid , C. M. Sanderson , Genomics 2006, 88, 333. 16730941 10.1016/j.ygeno.2006.04.003

[45] M. Gros , E. Segura , D. C. Rookhuizen , B. Baudon , S. Heurtebise‐Chrétien , N. Burgdorf , M. Maurin , E. A. Kapp , R. J. Simpson , P. Kozik , J. A. Villadangos , M. J. M. Bertrand , M. Burbage , S. Amigorena , Cell Rep. 2022, 40, 111205. 35977488 10.1016/j.celrep.2022.111205 PMC9396532

