# Supplementary material for: HNEAP Regulates Necroptosis of Cardiomyocytes by Suppressing the m5C Methylation of Atf7 mRNA
Source: Adv Sci (Weinh). 2023 Oct 23;10(34):2304329. doi: 10.1002/advs.202304329 (PMC10700171; doi:10.1002/advs.202304329)

ADVANCED SCIENCE
Open Access

Supporting Information

for *Adv. Sci.*, DOI 10.1002/advs.202304329

HNEAP Regulates Necroptosis of Cardiomyocytes by Suppressing the m^5C Methylation of Atf7 mRNA

Kai Wang, Fu-Hai Li, Lu-Yu Zhou, Xue-Mei Zhao, Xiang-Qian Gao, Cui-Yun Liu, Xin-Min Li, Xin-Zhe Chen, Yan Zhao, Xue-Li Cheng, Rui-Quan Wang, Rui-Feng Li, Yu-Hui Zhang, Fei Gao, Jin-Wei Tian* and Kun Wang**

SUPPLEMENTARY MATERIAL

Myocardial infarct size measurement. After reperfusion or sham treatment, 1.0% Evans blue dye (EBD) (Sigma, St. Louis, E2129, Missouri, USA) was injected into the coronary circulation of the mice to delineate the myocardial perfusion region. After taking out the heart, wash away the remaining blood and Evans blue dye and slice directly. Dye with 1-2% 2,3,5-triphenyl tetrazolium chloride (TTC) (Sigma, St. Louis, T8877, Missouri, USA) solution at 37℃ in the dark for 20min, then fix with 4% paraformaldehyde (Biosharp, BL539A, China) and take photos with a camera (Nikon-D5600). The infarct area was analyzed by ImageJ (NIH, Boston, USA).

Evans Blue Dye Assays. Mice were injected with 1% EBD at a dose of 100mg/kg through tail vein injection. After 24h, the mice were operated on with I/R or Sham operation, and the hearts were taken out and placed in the Tissue-TekOCT compound for rapid freezing. Using Leica cryostat for frozen sections (7nm), the frozen sections were anti-stained with wheat germ agglutinin (WGA) conjugate (AAT Bioquest, USA) in the ratio of 1:200, and the staining results were observed by confocal fluorescence microscope (Leica, TCS SP8) to quantify EBD positive cells.

Propidium iodide (PI) staining. After being treated with H/R, the cells were washed with PBS and stained with 30μg/ml PI (Solarbio, Beijing, China) at 37℃ for 30 min under dark conditions. After washing 3 times, 4% paraformaldehyde was fixed at

room temperature for 15-25min. Washing again 3 times with PBS, then the cells were anti-stained with 4-diamino-2-phenylindole (DAPI, Solarbio, Beijing, China) solution (1μg/ml) and observed under the fluorescence microscope (Nikon ECLIPSE Ti-S). The number of PI-positive nuclei was counted and quantified.

Lactate dehydrogenase (LDH) activity assay. LDH assay kit (Solarbio, Beijing, China) was used to determine the activity of LDH released from cell culture medium according to the instructions. After adding all the reagents according to the steps, mix them well, let them stand at room temperature for 3-5min, and use the Microplate reader (Synergy Neo2, BioTek, USA) to measure the absorbance value at the wavelength of 450nm. LDH activity was calculated according to beer's law.

Terminal deoxynucleotidyl transferase mediated dUTP nick end labeling (TUNEL) assay. TUNEL assay kit (Yeasen, Shanghai, China) was used to determine the apoptosis according to the instructions. Cell slides or tissue sections were fixed with 4% paraformaldehyde and permeabilized using 0.1% Triton X-100. After cleaning the slides with PBS, they were incubated with 1×Equilibration buffer for 30min. The cells were incubated with Terminal deoxynucleotidyl transferase (TdT) hybridization solution for 1h at 37°C in the dark. After the slides were cleaned, nuclei were labeled with DAPI.

Transfection of HNEAP agomir and antagomir. All HNEAP agomir or antagomir and their negative controls were obtained from GenePharma (Shanghai, China). Agomir or antagomir of HNEAP was transfected into cardiomyocytes using Lipo8000^{TM} Transfection Reagent (Beyotime, C0533, Shanghai, China). The HNEAP agomir sequence is 5'-UGAGGUUACUGAUGCAAUAUUUUUGAAGGG-3'. The HNEAP antagomir (anta) sequence is 5'-CCCUUCAAAAAUAUUGCAUCAGUAACCUCA-3'. And their negative control (NC and anta-NC) sequence is 5'-CAGUACUUUUGUGUAGUACAA-3'.

RNA interference (RNAi). Small interfering RNA (siRNA) oligonucleotides specific for Chmp2a and NC were purchased from GenePharma (Shanghai, China). Chmp2a siRNA (si-Chmp2a), 5'-AUGAUCUUCUGGAUCUGGGTT-3', Chmp2a-siRNA negative control (si-NC), 5'-ACGUGACACGUUCGGAGAATT-3'. Transfection of siRNAs was performed using Lipo8000^{TM} Transfection Reagent (Beyotime, C0533, Shanghai, China) according to the manufacturer's instructions.

RNA pull down. Cardiomyocytes transfected with biotinylated HNEAP and NC (GenePharma, Shanghai, China) were incubated in a lysis buffer containing protease inhibitor and RNA inhibitor. Then 10% volume of the lysis mixture was used as input, and 90% volume of the mixture was incubated with streptavidin magnetic beads (Biomake, Shanghai, China). Add 1/3 volume of 4×loading buffer to the collected

sample and boil it for 10min. After SDS-PAGE gel electrophoresis, the pull-down materials of HNEAP and NC were collected and analyzed by LC-MS/MS.

m^5C-IP-qPCR. To quantify the specific gene level of m^5C-modification, total RNA was extracted from cardiomyocytes or mouse hearts. Then the total RNA was fragmented, the RNA sample (10% of total volume) was used as the input control and the rest of the RNA sample was incubated with m^5C-antibody-conjugated beads in immunoprecipitation buffer containing RNase inhibitor at 4°C for 2h. The m^5C-containing RNA sample was eluted from the beads. qRT-PCR was performed with gene-specific primers for both the input control and m^5C-immunoprecipitated samples.

Nuclear and cytoplasmic RNA extraction. Collect cardiomyocytes and add CEB-A mixed solution containing DTT and protease inhibitor. The cells were completely resuspended and incubated on ice for 10 minutes. Add precooled CEB-B to the test tube. 16000g, centrifuged for 5 minutes. Take the supernatant as the cytoplasmic extract. After the precipitation was suspended with NEB, the supernatant was taken by vortex centrifugation as the nuclear extract. The whole process was operated in a low temperature environment, and the obtained samples were extracted by TRNpure Trizol Reagent or stored at -80°C.

Fluorescence in situ hybridization (FISH). HNEAP in cardiomyocytes can be labeled by specific fluorescent probes, and all steps are carried out following the instructions of the FISH Kit (gene Pharma, Shanghai, China). After 4% paraformaldehyde fixation and 0.1% Triton X-100 permeabilization, cardiomyocytes were dehydrated and dried in different concentrations of ethanol solution. Then add the hybridization buffer containing the fluorescent probe preheated at 37°C in advance, denature it at 73°C, and incubate it at 37°C in dark conditions overnight. After DAPI labeling, the distribution of HNEAP was observed under a confocal microscope.

2'-O-methylation at the 3'end. RTL-P is used to detect 2'-O-methylation at the 3' end, and the small RNA samples were extracted using mirVanamicroRNA isolation kit (Ambion, AM1556, USA). T4-RNA ligase (Takara, 2050A, Beijing, China) can connect 3' RNA adapter with the small RNA. In the presence of anchored or unanchored RT primers, the ligated products were reverse-transcribed using dNTPs at a high (50μM) or low (0.5μM) concentration, respectively. The product was amplified by PCR with specific primers and detected in agarose gel.

SUPPLEMENTAL FIGURE LEGENDS

Supplementary Figure 1. Identification of HNEAP in cardiomyocytes with H/R stimulation. (a and **b)** The expression levels of highly upregulated or downregulated piRNAs in H/R induced cardiomyocytes determined by qPCR (n=3 independent experiments). **(c)** qPCR result showing the expression level of DQ691316 in different cell types isolated from the mice hearts (myocytes, fibroblasts) (n=6 independent experiments). **(d-f)** Cardiomyocytes were subjected to H/R at indicated time. **(d)** Quantitative analysis of the percentage of necroptotic cardiomyocytes (n=5 independent experiments). **(e)** The representative western blot showing the expression of RIPK1 and RIPK3. **(f)** The expression level of HNEAP was detected by qPCR (n=6 independent experiments). Data are presented as Mean ± SD. Two-way ANOVA test (a, b), two-sided Student's t-test (c), One-way ANOVA test (d, f).

Supplementary Figure 2. Effects of HNEAP on cardiomyocyte apoptosis. (a, b) Cardiomyocytes were transfected with HNEAP agomir or antagomir (anta) and negative control (NC or anta-NC) for 24 h and then cells were treated with H/R. Quantitative analysis of the percentage of apoptotic cells (n=5 independent experiments). **(c)** Representative images of WT and HNEAP-KO mouse hearts. Bar=2mm. **(d)** Heart weight-body weight (HW/BW) ratio of WT or HNEAP-KO mouse hearts (n=5 mice per group). **(e)** Cardiac function (FS%) of WT or HNEAP-KO mouse hearts was measured by echocardiography (n=5 mice per group).

(f) Schematic overview of the experimental procedure. **(g)** WT and HNEAP-KO mouse hearts were stained with TUNEL to determine cell apoptosis. Quantitative analysis of the percentage of apoptotic cardiomyocytes (n=5 mice per group). Data are presented as Mean ± SD. One-way ANOVA test (a, b), two-sided Student's t-test (d, e), two-way ANOVA test (g).

Supplementary Figure 3. HNEAP binds to DNMT1. (a and **b)** A scheme showing the detection of HNEAP binding proteins using biotinylated HNEAP pull-down assay in cardiomyocytes. **(c)** qPCR analysis showing the mRNA level of DNMT1 in HNEAP-KO and WT mouse hearts (n=6 mice per group). **(d)** Representative western blot showing expression of DNMT1 in HNEAP-KO and WT mouse hearts. **(e)** qPCR analysis showing the mRNA level of DNMT1 in cardiomyocytes treated with or without HNEAP agomir or agomir-NC (NC) (n=6 independent experiments). **(f)** Representative western blot showing expression of DNMT1 in cardiomyocytes treated with or without HNEAP agomir or NC. Data are presented as Mean ± SD. Two-sided Student's t-test (c) or one-way ANOVA test (e).

Supplementary Figure 4. MeRIP-qPCR assay and qPCR assay of m^5C differentially methylated genes in HNEAP-KO and WT mouse hearts. (a and **c)** qPCR result showing the expression level of differential genes in HNEAP-KO and WT mouse hearts (n=3 independent experiments). **(b)** MeRIP-qPCR result showing the m^5C modification level of differentially methylated genes in HNEAP-KO and WT

mouse hearts (n=3 independent experiments). Data are presented as Mean ± SD. Two-way ANOVA test (a, b, c).

Supplementary Figure 5. The effects of Atf7 knockdown on cardiomyocyte apoptosis. (a-c) Cardiomyocytes were transfected with sh-Atf7 or its negative control (sh-NC). **(a)** Expression levels of Atf7 mRNA in cardiomyocytes (n=3 independent experiments). **(b)** Quantitative analysis of the percentage of necroptotic cardiomyocytes (n=5 independent experiments). **(c)** The activity of LDH in cardiomyocytes (n=3 independent experiments). **(d)** The representative western blot showing the expression of RIPK1 and RIPK3 in cardiomyocytes after transfection with sh-Atf7 or sh-NC and treated with H/R. **(e)** Apoptosis was determined by the TUNEL staining. DAPI indicates Nucleus. Bar=25μm. **(f)** Quantitative analysis of the percentage of apoptotic cells (n=5 independent experiments). Data are presented as Mean ± SD. One-way ANOVA test (a-c, f).

Supplementary Figure 6. HNEAP promotes Atf7 expression and cardiomyocyte necroptosis by DNMT1. (a) Cardiomyocytes were transfected with HNEAP antagomir (anta) or its negative control (anta-NC) for 24 h. The protein levels of ATF7 were detected by western blot assay. **(b)** After transfected with anta or anta-NC, the cells were treated with H/R. The protein levels of Atf7 were detected by western blot assay. **(c-e)** The cardiomyocytes were infected with DNMT1 shRNA (sh-DNMT1)

or its negative control (sh-NC) adenovirus and transfected with anta, and then were treated with H/R. **(c)** The protein levels of Atf7 were detected by western blot assay. **(d)** Quantitative analysis of the percentage of necroptotic cardiomyocytes (n=5 independent experiments). **(e)** The activity of LDH in cardiomyocytes (n=6 independent experiments). Data are presented as Mean ± SD. One-way ANOVA test (d, e).

Supplementary Figure 7. Knockdown of Chmp2a induced cardiomyocyte necroptosis. (a-c) Cardiomyocytes transfected with Chmp2a-siRNA (si-Chmp2a) or its negative control (si-NC). **(a)** The protein levels of Chmp2a were detected by western blot assay. **(b)** Quantitative analysis of the percentage of necroptotic cardiomyocytes (n=5 independent experiments). **(c)** The activity of LDH in cardiomyocytes (n=3 independent experiments). **(d)** The representative western blot showing the expression of RIPK1 and RIPK3 in cardiomyocytes after infected with sh-Atf7 or sh-NC and transfected with HNEAP. **(e)** The activity of LDH in cardiomyocytes after transfected with sh-Atf7 or sh-NC and transfected with HNEAP (n=3 independent experiments). **(f)** Cardiomyocytes were transfected with si-Chmp2a or si-NC after HNEAP knockdown, and treated with H/R. Quantitative analysis of the percentage of necroptotic cardiomyocytes (n=5 independent experiments). Data are presented as Mean ± SD. One-way ANOVA test (b, c, e, f).

Supplementary Figure 8. Model of HNEAP function in cardiomyocytes

necroptotic signaling. HNEAP participates in the regulation of cardiac necroptosis by targeting DNMT1/ATF7/CHMP2A pathway. In our model, HNEAP inhibits DNMT1-mediated m^5C methylation of Atf7 mRNA transcript, which leads to an increase of Atf7 expression, reduced Atf7-antagonistic Chmp2a expression and thus leads to cardiomyocytes necroptosis.

Supplementary Figure 1

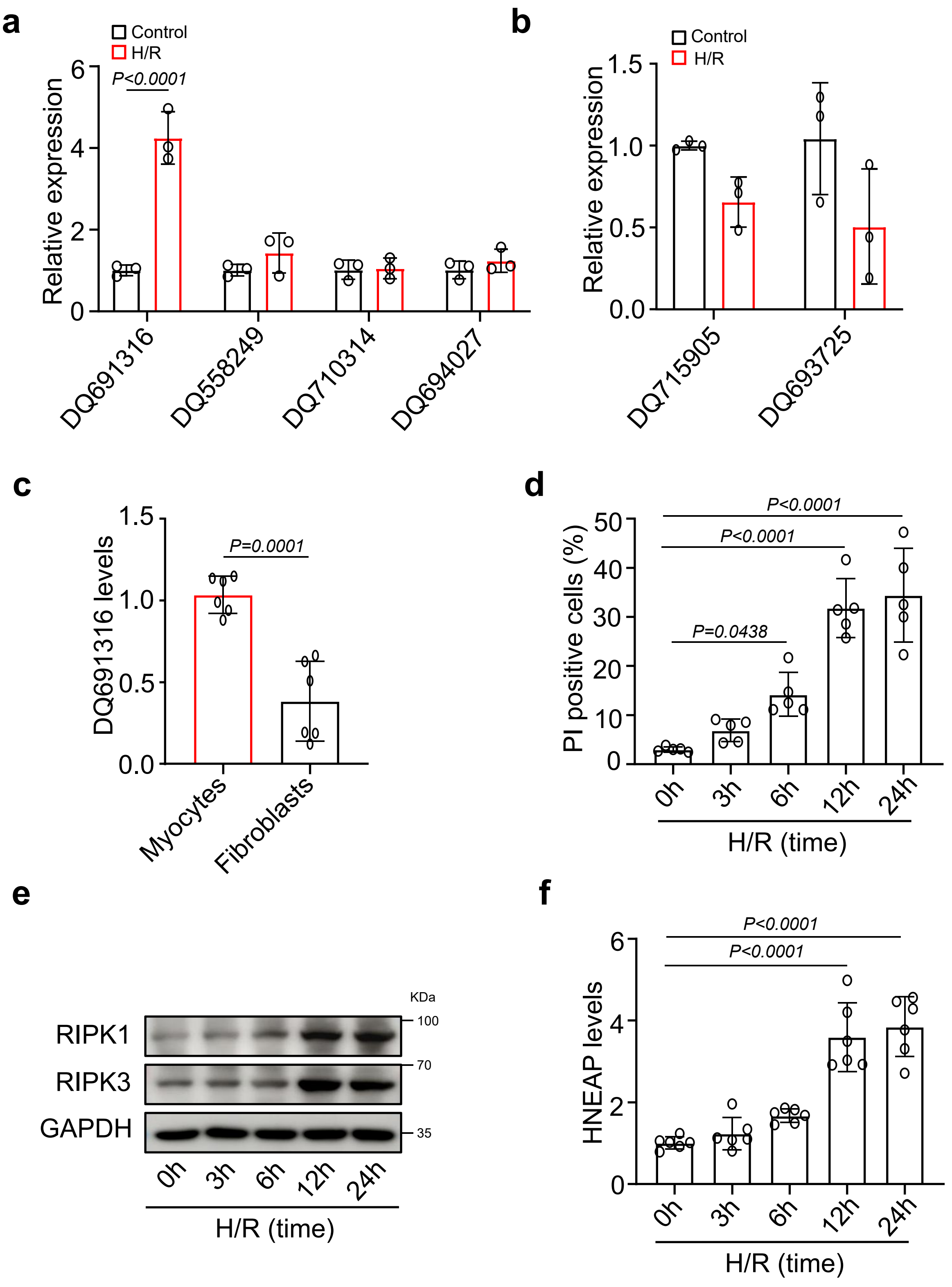

Supplementary Figure 2

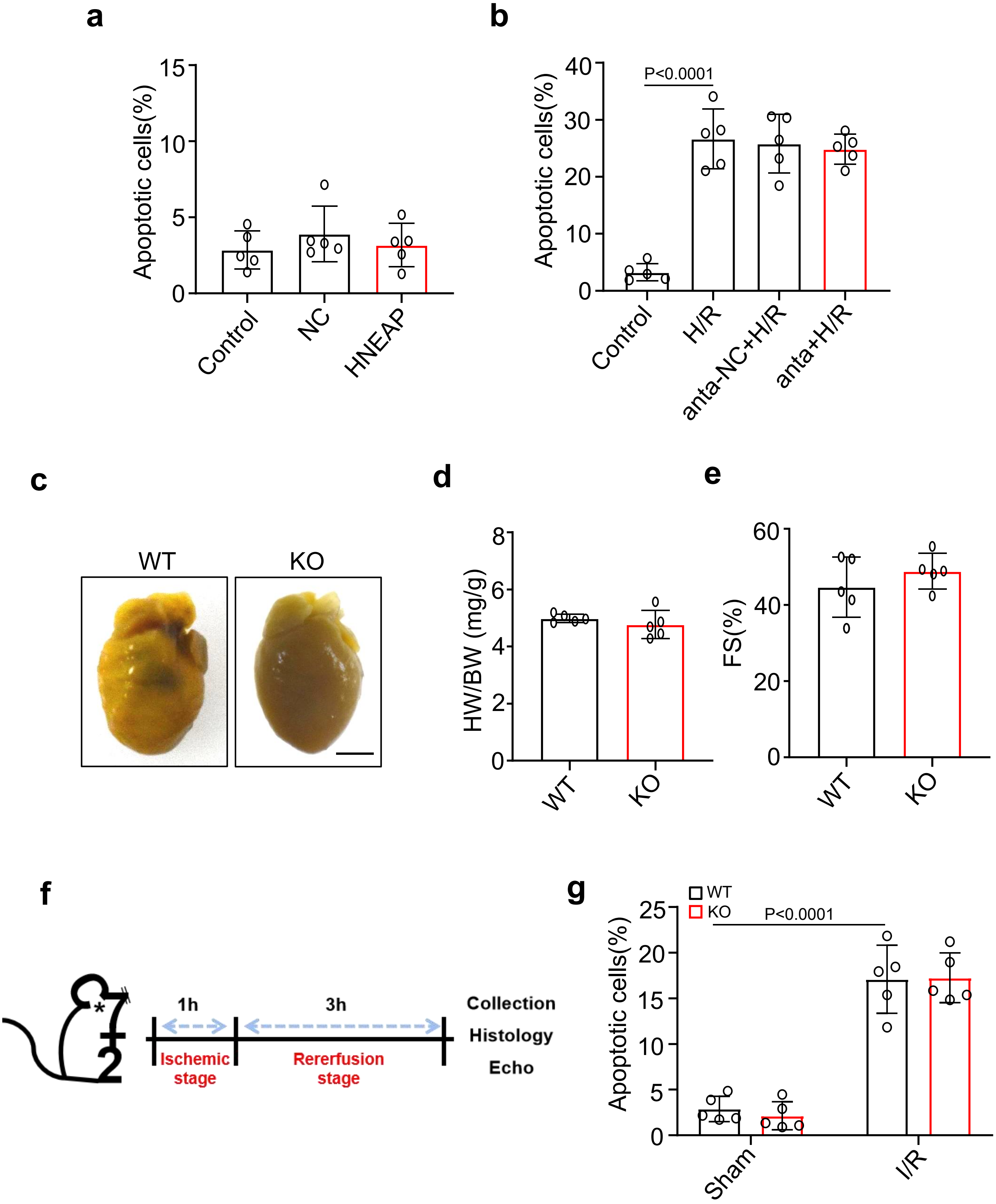

Supplementary Figure 3

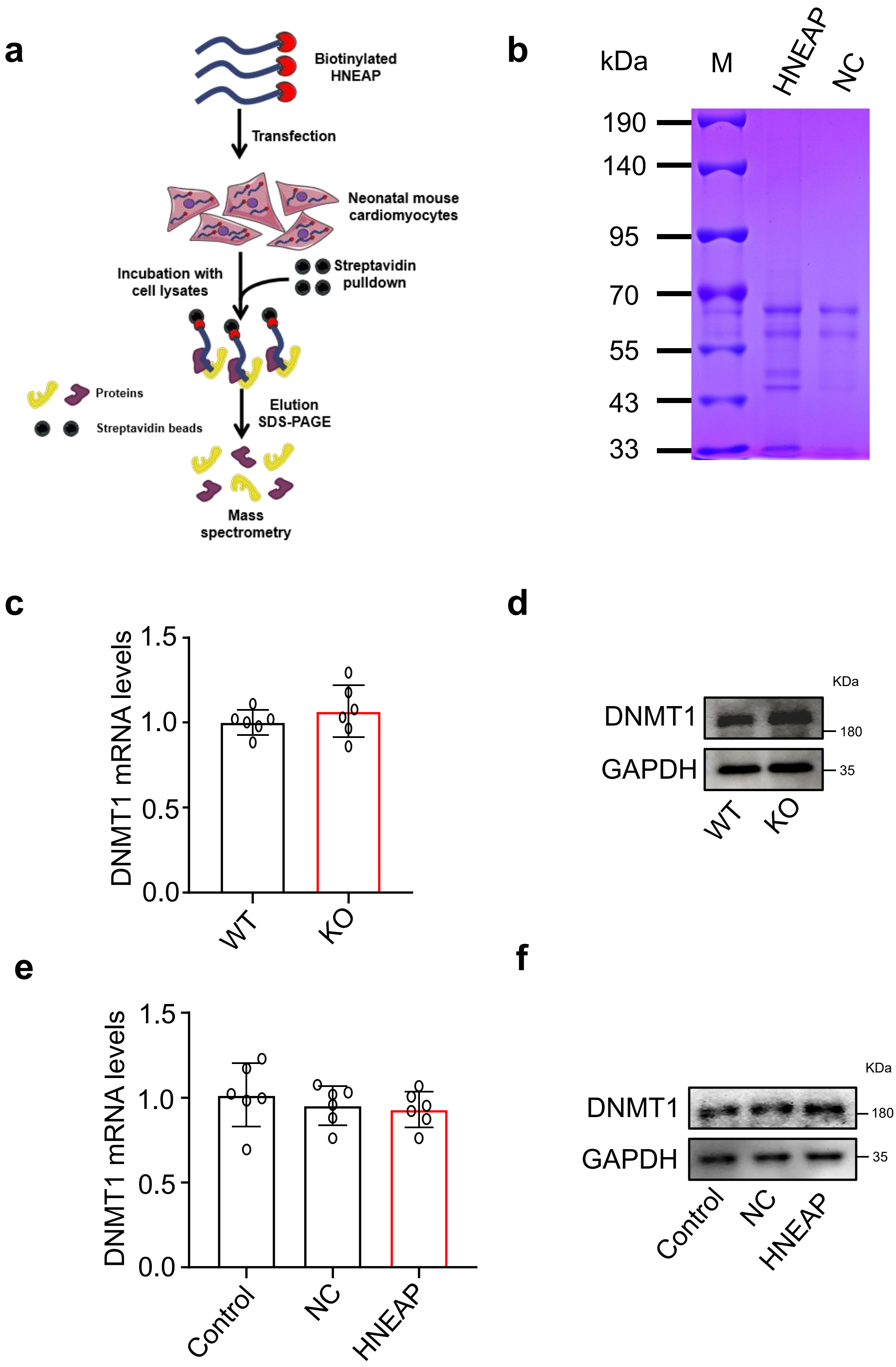

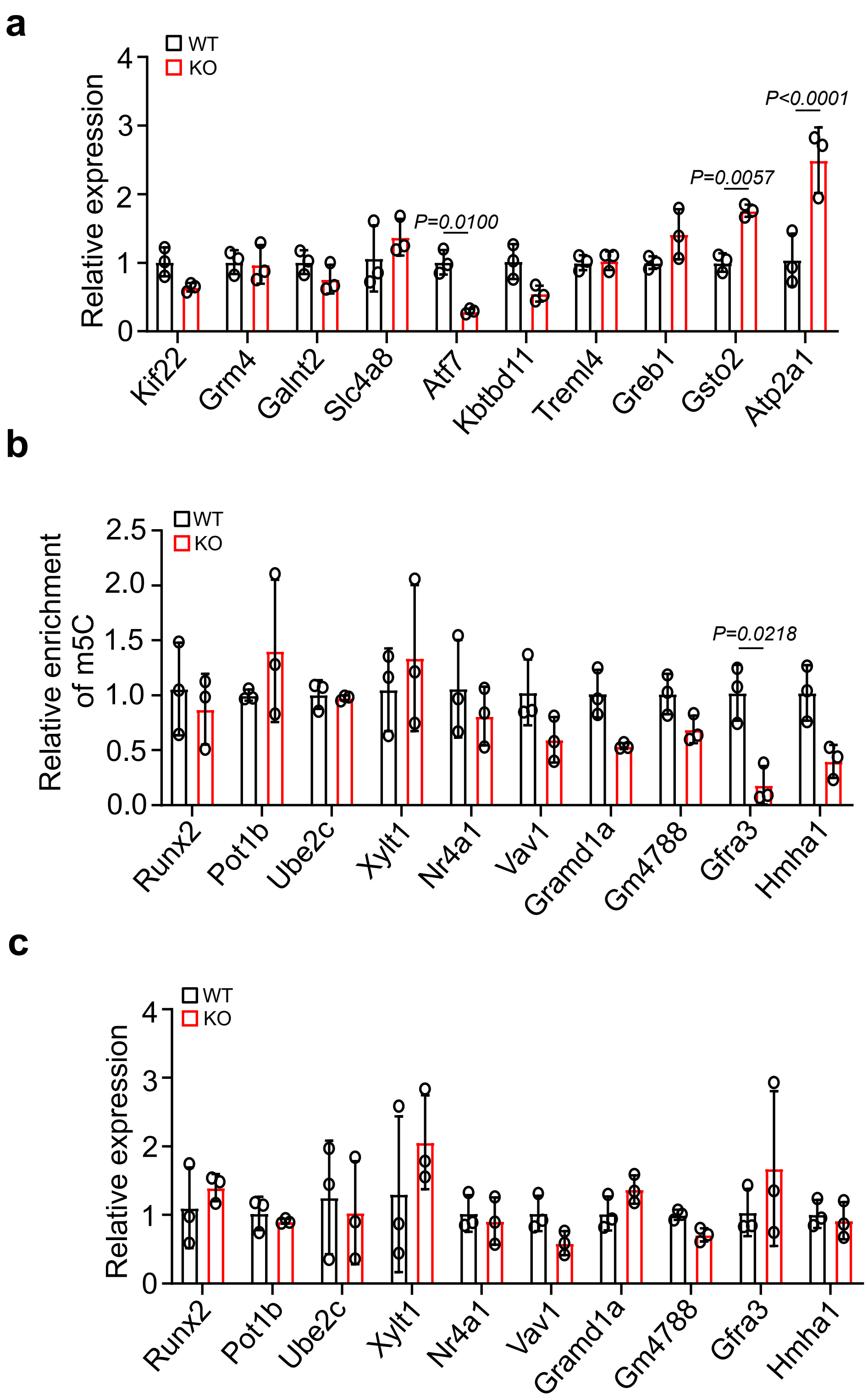
a
WT
KO
Relative expression
P=0.0100
P=0.0057
P<0.0001
Kif22
Grm4
Galnt2
Slc4a8
Atf7
Kbtbd11
Treml4
Greb1
Gsto2
Atp2a1
b
WT
KO
Relative enrichment of m5C
P=0.0218
Runx2
Pot1b
Ube2c
Xylt1
Nr4a1
Vav1
Gramd1a
Gm4788
Gfra3
Hmha1
c
WT
KO
Relative expression
Runx2
Pot1b
Ube2c
Xylt1
Nr4a1
Vav1
Gramd1a
Gm4788
Gfra3
Hmha1

Supplementary Figure 5

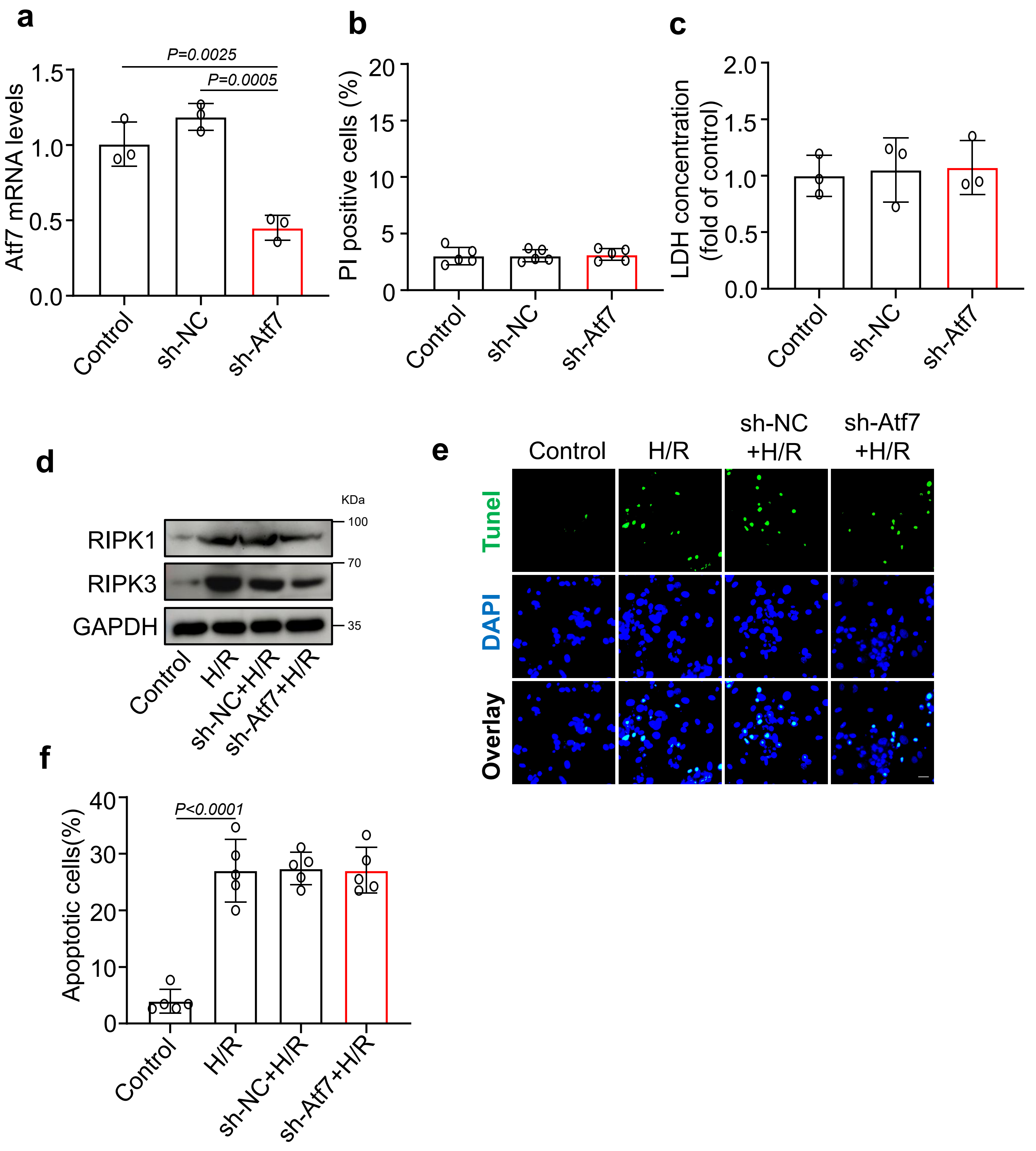

Supplementary Figure 6

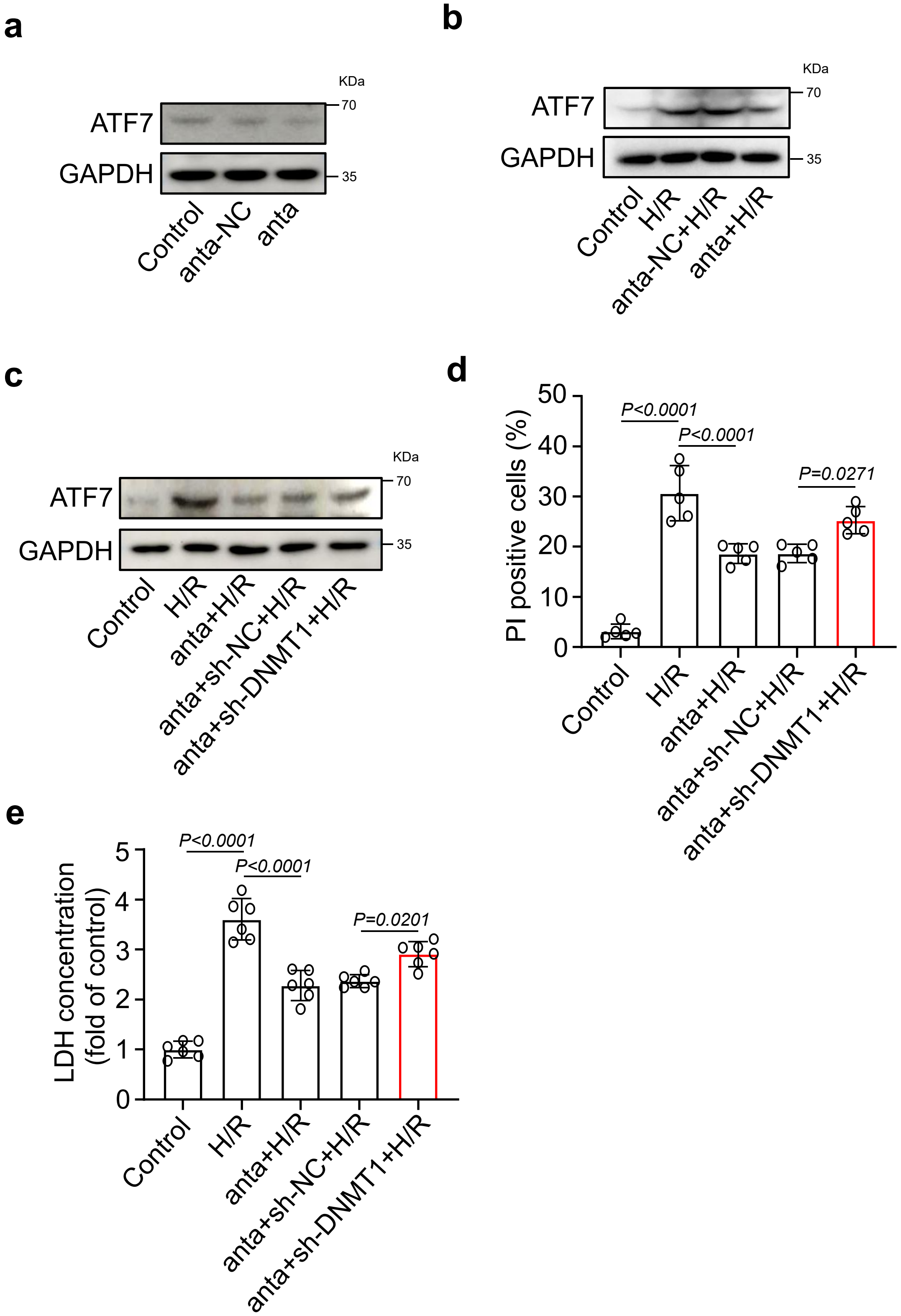

Supplementary Figure 7

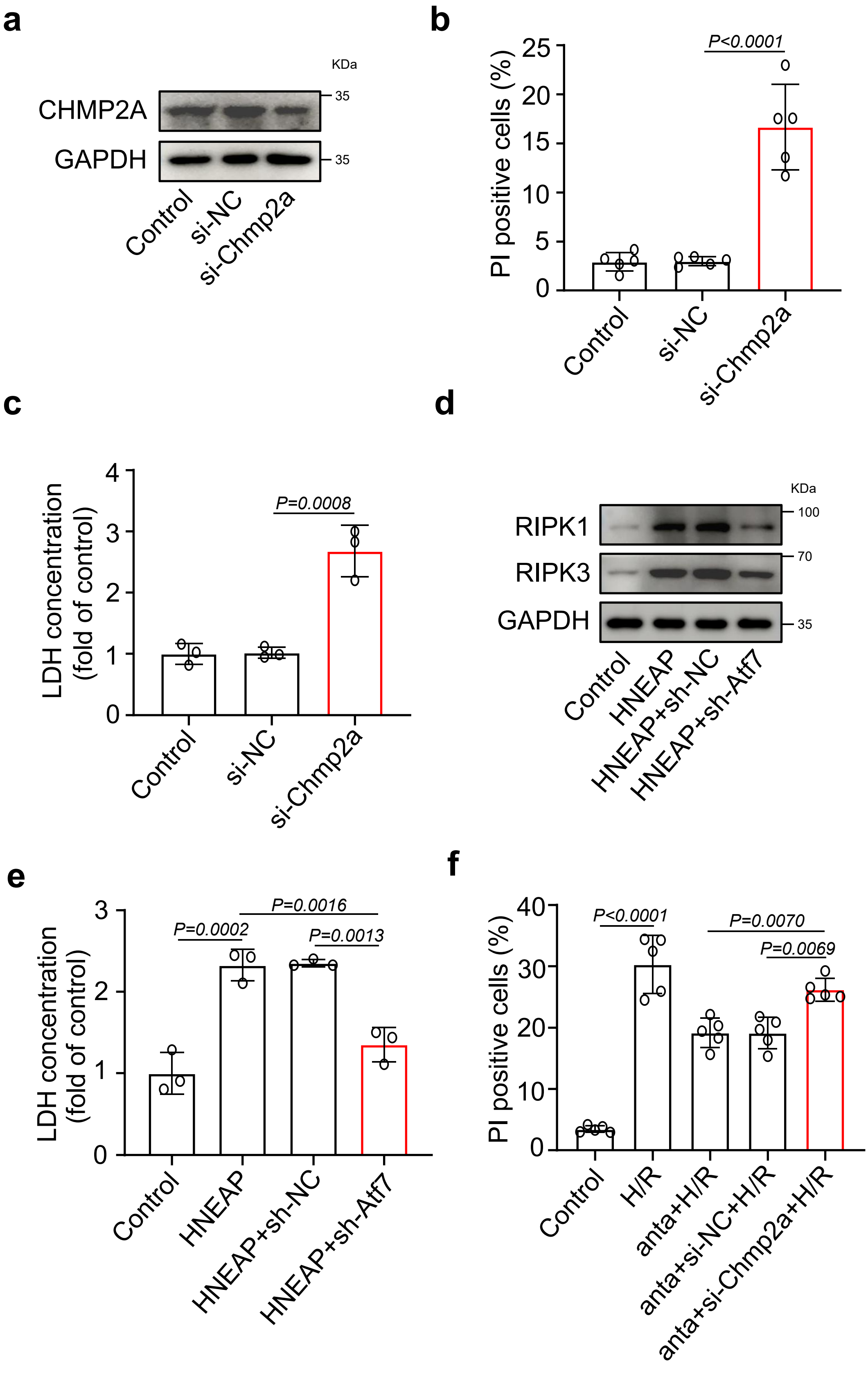

Supplementary Figure 8

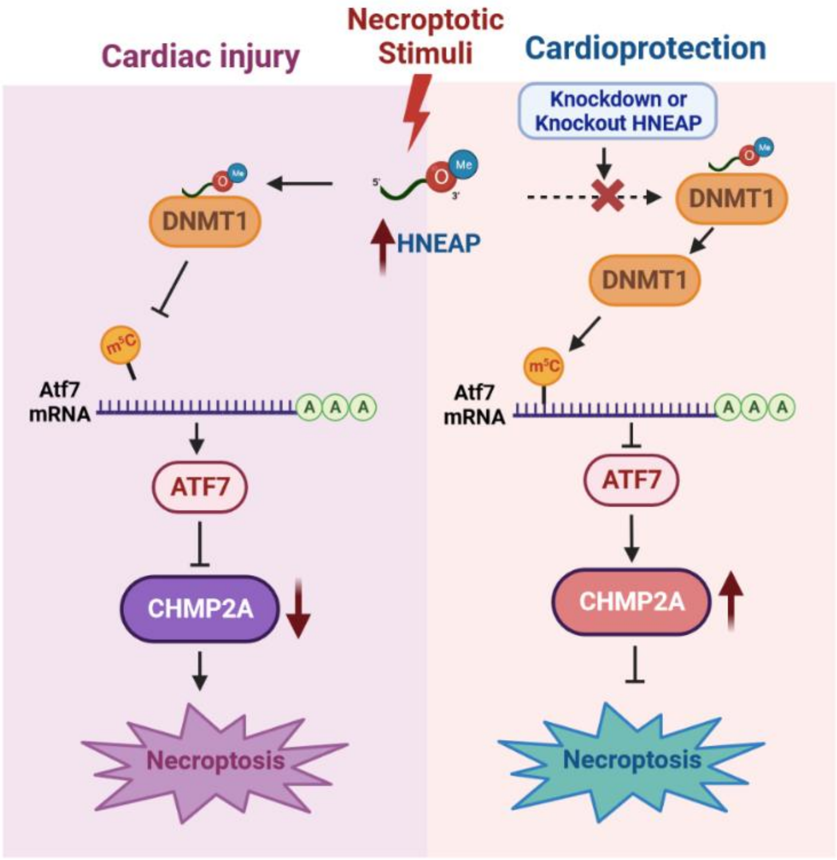

Supplement: Supplementary file 1 — Supporting Information [file ADVS-10-2304329-s005.pdf]
